# An iris-like pore for protein import into peroxisomes

**DOI:** 10.64898/2026.10.23.761396

**Published:** 2026-10-28

**Authors:** Dingwei He, Yuan Gao, Tom A. Rapoport

**Affiliations:** 1 Department of Cell Biology, Harvard Medical School, Boston, MA 02115, USA.; 2 Howard Hughes Medical Institute, Harvard Medical School, Boston, MA 02115, USA.; 3 Current address: Lyterian Therapeutics, South San Francisco, CA 94080, USA

## Abstract

Peroxisomes are ubiquitous, membrane-bound organelles that house essential metabolic enzymes imported from the cytosol in a folded and sometimes oligomeric state. Mobile receptors, such as PEX5, ferry cargo through a tyrosine/glycine (YG)-rich selective phase formed by PEX13 within the membrane, but how folded proteins of different sizes cross the membrane remains unknown. Here we report structures of the channel that mediates cargo import. Twelve PEX13 molecules assemble into a pore formed by a ring of tilted amphipathic helices in alternating orientations. Hydrophobic faces contact the lipid bilayer, whereas hydrophilic faces line the pore containing the YG phase. Pairs of oppositely oriented helices can slide relative to neighboring pairs and thereby alter the helix tilt angle, enabling iris-like dilation of the pore from ~52 Å in the resting state to ~70 Å, sufficient to accommodate the PEX5 receptor carrying small cargo. Translocation of the largest cargo requires further widening to ~100 Å, a state supported by AlphaFold modeling and disulfide crosslinking. Our findings suggest that the channel provides an adaptive conduit for folded proteins of varying sizes, a mechanism distinct from other known modes of protein translocation.

Peroxisomes are ubiquitous organelles that consist of a single membrane enclosing a lumen (matrix) packed with proteins involved in various metabolic pathways, including fatty acid oxidation and detoxification of reactive oxygen species. Peroxisomes are vital for human health; peroxisomal disorders lead to debilitating and often fatal diseases, notably the Zellweger spectrum disorders (for review, see^1–4^).

Peroxisomes import their matrix proteins post-translationally from the cytosol (for review, see^1,2,5–9^). Remarkably, these proteins are translocated across the membrane in a folded or even oligomeric state, unlike proteins destined for the ER, mitochondria, or chloroplasts, which must be unfolded for import. How folded proteins of different sizes traverse the peroxisomal membrane remains unknown.

Peroxisomal protein import is mediated by soluble receptors that recognize cargo in the cytosol and deliver it to the matrix. Most imported proteins carry a C-terminal peroxisome-targeting signal (PTS1), typically the sequence SKL or a close variant^10^, recognized by the tetratricopeptide-repeat (TPR) domain of the receptor PEX5^11^. The cargo-bound receptor is recruited to the peroxisomal membrane by the “docking complex”, which contains the conserved membrane proteins PEX13 and PEX14^12^. The receptor then ferries bound cargo through the peroxisomal membrane all the way into the matrix^13^. Translocation occurs through a nuclear pore-like conduit formed by PEX13^14,15^. PEX13 contains a conserved tyrosine/glycine-rich (YG) domain that resembles the phenylalanine/glycine (FG) repeats of nucleoporins^16–18^. The YG domains of multiple PEX13 molecules form a meshwork within the membrane, generating a selective phase that excludes inert molecules above a size threshold while allowing receptors to partition into it through conserved aromatic motifs in their unstructured N-terminal regions^14^. After cargo delivery, the receptor is recycled to the cytosol by a retro-translocon comprising a membrane-embedded ubiquitin ligase complex, followed by extraction by a hexameric ATPase^19–22^. A subset of matrix proteins carries an N-terminal PTS2 signal that binds to both a receptor and the adaptor PEX7^23,24^. This trimeric complex traverses the YG phase to deliver cargo to the matrix; the receptor is then recycled by the retro-translocon, while PEX7 returns independently to the cytosol through the YG conduit^25^ and is extracted by PEX39^25,26^.

The central unresolved question concerns the architecture of the import conduit itself. Recent work suggested that PEX13 forms the translocon for peroxisomal import^14^. PEX13 has only two conserved features: the unstructured YG domain and a long amphipathic helix (AH) (Fig. 1a). The YG domain is preceded by an additional disordered region that is only required for PTS2 import^25–27^. In many organisms, PEX13 also has a predicted C-terminal transmembrane (TM) segment and an SH3 domain (Fig. 1a), but these are absent in plants and algae^8^. Protease-protection experiments indicate that PEX13 adopts dual topology in the membrane: half the molecules have their C terminus facing the cytosol and the other half facing the matrix^14^. Although dual topology is unusual among membrane proteins, it allows the YG domains of both PEX13 populations to meet within the plane of the membrane and form a meshwork. How this YG phase is suspended within the membrane and insulated from the surrounding lipid remains unknown.

Here we report cryo-EM structures of the peroxisomal protein import channel. Twelve PEX13 molecules assemble into a pore ring in which amphipathic helices form the channel wall around the YG phase. Experimental structures of 52 Å and 70 Å pores, together with a predicted 100 Å pore supported by disulfide crosslinking, show that helix pairs slide relative to one another, allowing the channel to dilate like an iris and accommodate folded cargo of different sizes.

## Cryo-EM structures of the wild-type PEX13 channel

To obtain a structure of the PEX13 channel, we expressed the wild-type human protein with a streptavidin-binding peptide (SBP) tag at the C terminus in *Pichia pastoris* cells (Fig. 1a). A membrane fraction was isolated, the protein was solubilized in the detergent digitonin and purified by binding to streptavidin beads followed by size-exclusion chromatography (SEC) (Fig. 1b). The protein eluted as a sharp peak at high molecular weight. Analysis of the peak fraction by SDS-PAGE and Coomassie blue staining revealed a single band of expected size (Fig. 1b). PEX13 maintains its oligomeric state after detergent solubilization and purification, as shown by introducing two cysteines into either the YG or AH domain and adding an oxidant. In both cases, a ladder of disulfide-linked adducts was observed, which largely disappeared after reduction of the disulfides with dithiothreitol (DTT) (Fig. 1c).

The purified wild-type (WT) protein was used for cryo-EM single-particle analysis. 2D classification of the particles showed side views in which the detergent micelle is bordered by several dots (arrowheads in Fig. 1d; **Extended Data Fig. 1a-c**). These should correspond to the SH3 domains, as PEX13 does not possess any other globular domain outside the membrane. Thus, in agreement with the predicted dual topology in intact membranes^14^, some PEX13 molecules have their SH3 domain on one side of the detergent micelle, while others have it on the opposite side. The side views also indicate that the dataset contains particles of different sizes (Fig. 1d).

Consistent with the heterogeneity of the particles, we obtained different density maps from the dataset with 6-fold symmetry imposed during data processing (**Extended Data Fig. 2; Extended Data Table 1**). All reconstructions resulted in refined density maps with an overall resolution of ~6.2 Å (Fig. 1e; f; **Extended Data Fig. 3a**). The maps show a ring of twelve left-tilted helices surrounding an almost circular pore. In two maps, the pore diameter is ~70 Å, and in the third ~52 Å. The two density maps with the larger pore size are almost identical; the only difference is additional density at the end of the helices (**Extended Data Fig. 3a**). Because of the limited resolution, no helical turns or side chain density were discernible.

The tilt angle of the AHs (defined as deviation from the membrane normal) is smaller for the ~52 Å pore than the ~70 Å pores (53° versus 58°) (Fig. 1e; f). The channels also differ in their height (47 Å versus 44 Å). The SH3 domains were seen at low contour level (**Extended Data Fig. 3b**), but seem to be flexible. The TMs might be responsible for the weak density seen at the periphery of the pore (**Extended Data Fig. 3c**).

Molecular models for the ~52 Å and ~70 Å channels were derived with the help of AlphaFold^28^. Using 12 copies of the AHs, AlphaFold3 predicts channels of different diameters with low confidence scores (ipTM 0.2–0.3). Including 12 copies of the TMs in the analysis, resulted in improvement of the predictions, although AlphaFold3 randomly converged on channels of either ~52 Å or ~70 Å diameter (**Extended Data Fig. 4a**). In both cases, the AHs were predicted with much higher confidence than the TMs and formed pores that fit well into the experimental density maps (Fig. 1e–h). Models for the AHs start at the N-terminus of the AH (residue S116) and extend to residues 171 and 176 in the ~52 Å and ~70 Å channels, respectively (Fig. 1g; h). Residues 177 to 199 of the AH are invisible in all maps. A kink in the density map is seen at opposite ends in neighboring helices, consistent with the AlphaFold models in which AHs alternate in their membrane topology (**Extended Data Fig. 4a**), and with the placement of the SH3 domains to both sides of the channels (**Extended Data Fig. 3b**). Two AHs of opposite orientation form a unit that is repeated six times in the certain tilt angle around the ring, explaining why imposition of 6-fold symmetry was required for structure determination. When viewed perpendicular to the membrane plane, the helices are curved, forming blades as in an iris diaphragm (Fig. 1e; f). In both channels, the AHs have their hydrophobic faces oriented towards the lipid and their hydrophilic faces pointing towards the interior of the channel (Fig. 1i; j), supporting the validity of the AlphaFold predictions. Furthermore, all helix interfaces leave only small holes in the channel walls.

As helix polarity was difficult to assign from the density maps, we used *S. cerevisiae* PEX13 to confirm that neighboring AHs in the PEX13 channel have opposite membrane orientations. Endogenous yeast PEX13 with a single cysteine in the AH was fused at its C terminus to a 3C protease-cleavage site, followed by an SBP tag to enhance the size shift after proteolysis. An N-terminal FLAG tag was added for immunodetection (Fig. 2a).

Membranes were isolated from yeast cells and incubated first with the protease and then with an oxidant. When analyzed by reducing SDS-PAGE, two bands were seen (Fig. 2a), corresponding to the two orientations of the C terminus; the protease-resistant pool had the C terminus in the matrix, shielded by the membrane, as it could be cleaved only after membrane solubilization with detergent. Under oxidizing conditions, a single disulfide-linked band was observed after cleavage (Fig. 2a), which corresponds to an adduct of two PEX13 molecules with opposite orientations of their C termini. Analogous experiments with a cysteine introduced into the YG domain show three bands under oxidized conditions (Fig. 2a), as reported previously^14^. These bands correspond to PEX13 dimers in which the two molecules have their C terminus either both in the matrix, on opposite sides of the membrane, or both in the cytosol (Fig. 2b). Thus, in contrast to crosslinked AHs, crosslinked YG domains consist of PEX13 molecules of both orientations. These experiments indicate that neighboring AHs have opposite membrane orientations and support the dual-membrane topology model in Fig. 2b.

The YG domain is required for proper channel formation. When the YG domain was deleted, PEX13 eluted in SEC as a broader peak at lower molecular weight than the wild-type protein (**Extended Data Fig. 5a**). A similar result was obtained when all tyrosines of the YG domain were mutated to serines (**Extended Data Fig. 5b**). Disulfide crosslinking with two cysteines introduced into this mutant demonstrated that the protein no longer forms a ladder and thus has a defect in oligomerization (**Extended Data Fig. 5c**). The channel is therefore stabilized by the YG phase.

## Cryo-EM structure of an inactive PEX13 channel

We noticed that the AH contains an unusual concentration of acidic amino acid residues (see sequence alignment in **Extended Data Fig. 6**). Two of these residues are conserved in most organisms, and additional ones in a subset of species. To test whether the acidic residues are required for peroxisomal protein import, we used an engineered pathway in *S. cerevisiae* cells that generates a pigment when import is impaired^29^. Mutating only one of the three conserved acidic residues (E153, E167, E185) had no effect, but a double mutant (2E/A) showed reduced import activity and the triple mutant (3E/A) was essentially inactive (Fig. 3a). Mutating the equivalent three negatively charged residues in human PEX13 to alanines (E134A, D148A, D163A; 3ED/A mutant) did not affect the purification of the protein; like the wild-type protein, the mutant eluted in SEC as a sharp peak at high molecular weight (Fig. 3b). Together, these data suggest that the triple mutant forms an inactive channel.

The purified 3ED/A mutant was subjected to single-particle cryo-EM analysis. The side views in 2D averages showed that the heterogeneity of channel size was considerably reduced compared with the wild-type complex (**Extended Data Fig. 1d**). A map of 3.3 Å overall resolution could be obtained without imposing symmetry during refinement, but significant improvement was achieved after imposing 3-fold symmetry, resulting in a resolution of 2.8 Å (Fig. 3c; **Extended Data Table 1**). The final map shows a ring of 12 tilted AHs surrounding a central pore. The amino acid side-chain density was sufficient to model more than 60 residues of the AHs (Fig. 3d; **Extended Data Fig. 7a**). The tilt angle of the AHs (52°) and the pore diameter (~52 Å) are similar to those of the small wild-type channel (53° and ~52 Å, respectively). In contrast to the wild-type channels, which are formed from 6 copies of AH pairs, the mutant channel consists of 3 copies of four AH-containing units, a feature observed even without imposing symmetry during refinement. As before, the AHs alternate in their orientation around the ring, consistent with the dual topology in membranes and the placement of SH3 domains on both sides of the micelle (**Extended Data Fig. 1d**). The hydrophobic and hydrophilic faces of the AHs are again oriented outwards and inwards, respectively (Fig. 3e). The mutated negative charges all point into the pore (**Extended Data Fig. 8**). We speculate that the absence of charge repulsion in the 3ED/A pore disfavors channel widening.

Molecular models could also be built for 9 of the 12 TM segments (Fig. 3c; d; **Extended Data Fig. 7b**); the missing TM segments are likely flexible. The visible TM segments are positioned outside the pore and sit perpendicular to the membrane plane. They contact the outer, lipid-facing surface of the AH wall through hydrophobic residues. The interaction with a TM might be responsible for a kink of the AH near its N terminus. Some density seen in the unsharpened map inside the pore likely corresponds to the YG domains, arranged as sheets lining the hydrophilic faces of the AHs (**Extended Data Fig. 7c**).

## A large channel predicted by AlphaFold

The wild-type channel of ~70 Å pore size is too small to allow the import of some cargo (see Discussion). However, AlphaFold2 predicts a large channel of ~100 Å diameter with reasonable per-residue pLDDT scores and PAE plot (**Extended Data Fig. 4b**). The predicted channel contains highly tilted AHs (tilt angle ~70°) (Fig. 4a). The C-terminal regions of the AHs (residues 177 to 199), which are invisible in the smaller pores, extend the helices to form the channel wall. The channel height is reduced to 29 Å (Fig. 4a). As with the smaller pores, the hydrophobic and hydrophilic sides of the AHs are oriented outward and inward, respectively, and the helix interfaces leave only small holes in the channel wall (Fig. 4b), giving further credence to the prediction.

Channels of different pore sizes are also predicted for *S. cerevisiae* PEX13. AlphaFold3 predicts channels of ~52 Å and ~70 Å, and AlphaFold2 a channel of ~100 Å (**Extended Data Fig. 4c**). All predictions have low confidence scores, but the structures are similar to the corresponding predicted or observed channels of the human protein (**Extended Data Fig. 4a; b**; Fig. 1e; f). The predictions are further validated by the analysis of the interfaces of the AHs and crosslinking experiments (see below). Thus, peroxisomal import channels of different sizes seem to occur in evolutionarily distant organisms.

## An iris-like mechanism of pore size adjustment

The channel structures can be described as left-handed toroids. The left handedness is dictated by the chirality of the α-helices and the requirement that the hydrophobic sides face lipid. In all structures, each AH has two neighbors of opposite orientation. One of the two interfaces is constant and the other variable. The constant interface is the same in all channel structures of human PEX13 (Fig. 4c–e). It contains clusters of aromatic residues (Phe and Tyr) at the ends of both interacting helices (Phe130, Phe151, Tyr155, Phe158) and an aromatic residue at the center (Phe140). The aromatic residues lock the two helices together, so that they behave as a unit (fixed dimer). The amino acids in the constant interface are conserved between several different species (**Extended Data Fig. 6**).

The variable interface contains amino acids with small side chains, primarily Ala, Gly, Val, Thr and Ser (Fig. 4c–e). This interface allows neighboring helices to move relative to one another. Using residue A153 as reference (red star), the variable interface shifts by about one helix turn from the ~52 Å to the ~70 Å channel, and by additional ~ 3 turns to the ~100 Å channel (Fig. 4c–e). The 3ED/A mutant channel has two distinct variable interfaces on either side of the fixed dimer (interfaces 1 and 2), causing the observed 3-fold rotational symmetry (Fig. 4f). Interface 2 is almost identical to the single variable interface of the ~52 Å wild-type channel (Fig. 4c). The comparison of these two channels shows that AH dimers can slide longitudinally along the helical axis with minimal changes of their tilt angle and pore diameter.

As in mammals, all three *S. cerevisiae* channels have the same constant interface with interacting aromatic residues and distinct variable interfaces containing small side-chain amino acids (**Extended Data Fig. 9a-c**). These similarities again support the validity of the AlphaFold predictions. As expected from the sequence features of the interfaces, mutation of three residues in the constant interface into alanines (Y170A, H174A, F177A, YHF/A mutant), or conversion of one of the small residues (Ala172) in the variable interface to a bulky amino acid (Phe, A172F mutant) abolishes peroxisomal protein import in *S. cerevisiae* (**Extended Data Fig. 9d; e**).

The fixed dimers can undergo radial sliding along the variable interface to generate different tilt angles and corresponding pore sizes, as shown by superimposing these helices for the ~52 Å and ~70 Å channels, and for the ~70 Å and ~100 Å channels (Fig. 4f). As the pore widens from ~52 Å to ~100 Å, the rigid dimers slide past their neighbors around the ring, like the blades of a camera iris opening, moving farther from the pore’s center while tilting to lie flatter against the membrane (Fig. 1e;f; Fig. 4a; **Extended Data movie 1**). Whereas longitudinal sliding can occur with individual dimers, the radial change requires that all six dimers tilt in the same way. During sliding, the helices do not significantly rotate about their axis, thus maintaining the orientation of hydrophobic residues towards lipid. Channel widening is accompanied by a reduction of the membrane thickness surrounding the channel **(Extended Data movie 1**). The small and intermediate sized channels of ~52 Å and ~70 Å have hydrophobic belts of ~32–35 Å thickness (Fig. 1i; j), which corresponds to the normal hydrophobic core of a phospholipid bilayer. The largest channel of ~100 Å has a hydrophobic belt of ~22 Å (Fig. 4b), which would cause distortion of the phospholipid bilayer. and may therefore have to be stabilized by receptor and cargo.

## Helix sliding confirmed by disulfide crosslinking

To verify that the sliding of the AHs generates different pores sizes, we performed disulfide crosslinking experiments with native *S. cerevisiae* membranes. FLAG-tagged PEX13 with single cysteines at different positions of the AH was expressed in yeast and isolated membranes were treated with an oxidant. The samples were analyzed by nonreducing or reducing SDS-PAGE (Fig. 5a). The observed crosslinks with a cysteine at position 160 can be explained with all three channel structures, as this residue is located at the constant interface and is in disulfide-bonding distance to the same residue in the neighboring helix (Fig. 5b). Crosslinks indicative of the variable interface in the ~52 Å channel were observed with a cysteine at position 168 (Fig. 5b). Disulfide-bond formation with a cysteine at position 175 is consistent with the ~70 Å and ~100 Å channels, whereas crosslinks with position 180 can only be explained with the variable interface expected for the ~100 Å channel (Fig. 5b); this residue would be too distant from the same residue in a neighboring helix in the other channel structures (>12 Å). Only little crosslinking was seen with several cysteine positions picked as controls (**Extended Data Fig. 10a**).

Similar disulfide crosslinking experiments confirmed the different channels formed by purified human PEX13 (Fig. 5c; d). Position 141, located at the constant interface, is compatible with all three predicted channel structures. Crosslinks indicative of the variable interface in the ~52 Å channel were observed with a cysteine at position 149 (Fig. 5c; d). Position 156 is consistent with the ~70 Å and ~100 Å channels; and position 161 is compatible only with the ~100 Å channel. In both cases, a ladder was generated, although it was less pronounced with positions 141 and 161, suggesting that the ~100Å channel is less abundant. (**Extended Data Fig. 10b**). Together, these data confirm the different channels generated by yeast and human PEX13.

## Discussion

Our structures show that the peroxisomal import channel consists of twelve PEX13 molecules. The walls consist of a ring of tilted AHs in alternating orientations, with hydrophobic faces on the outside, contacting the lipid, and hydrophilic faces inside, surrounding the YG phase. The channel can adopt different pore sizes by an iris-like movement of the AHs.

The pore of ~52 Å is a resting state, into which the channel is locked when three conserved negative charges of the AH are mutated. This channel does not permit peroxisomal protein import in yeast, consistent with it being too narrow to allow passage of the C-terminal TPR domain of PEX5 (dimensions of ~40 × 60 Å^11^). Although peroxisomes are permeable to molecules smaller than ~600 Da^29,30^, a resting state with a dense YG meshwork inside the pore might be required to limit the exchange of metabolites between the cytosol and peroxisomes.

The ~70 Å channel would be just large enough to accomodate the TPR domain^31^ (**Extended Data Fig. 11a**). Monomeric matrix proteins bound to the TPR domain could also move through, as suggested by modeling the channel with a known structure of TPR-bound cargo in its interior^31,32^ (**Extended Data Fig. 11b**). In fact, if the TPR domain and cargo passed the pore one after the other, the ~70 Å pore could transport even the largest known monomeric matrix protein (~79 kDa^33^, corresponding to a spherical diameter of 57 Å), as well as oligomeric proteins known to be imported as monomers (72–75 kDa)^34–36^. Monomeric PTS2 cargoes bound to the adaptor PEX7 (32 × 45 Å) and a short receptor segment could also move through the ~70 Å channel. The YG domains would likely have to be displaced, but a similar situation is seen in the nuclear import of the HIV-1 capsid, which interacts with the FG repeats, but has a diameter that is almost identical to the width of the nuclear pore^37,38^.

The ~52 Å channel is likely the most stable state but may be in equilibrium with the ~70 Å channel, as both structures co-exist in the cryo-EM dataset. The spontaneous switching between the two states may be facilitated by both channels sitting in an undistorted lipid bilayer. Escape from the ~52 Å channel state requires the conserved acidic residues in the AH; they all point into the pore and are most tightly packed in the ~52 Å channel (**Extended Data Fig. 8**), possibly generating electrostatic repulsion that could facilitate channel widening.

Although the ~70 Å channel might be sufficient for some cargo, channel widening is certainly required for others. The predicted ~100 Å channel could accommodate the largest oligomeric proteins, such as the tetramer of isocitrate lyase (~256 kDa)^39^. It would also be needed for the observed import of 9 nm gold particles coated with PTS1 peptides^40^.

Although an experimental structure of the ~100 Å channel is currently unavailable, the AlphaFold-prediction is supported by (1) the hydrophobic and hydrophilic surfaces of the AHs pointing outwards and inwards, respectively, (2) the channel walls having only small holes; (3) the AHs sharing the constant interface with the experimentally observed channels, and (4) the variable interface being independently confirmed by disulfide crosslinking with intact membranes and purified protein. The ~100 Å channel is probably energetically unfavorable, as it would sit in a thinned and distorted lipid bilayer and has weaker AH interactions than the smaller channels. Although challenging, it will be important to test experimentally whether channel widening is induced when PEX5 and large cargo move through.

Although we have not directly visualized the transitions between the different channel states, they likely occur by a simple iris-like motion of the AHs. Two neighboring AHs of opposite orientation form a fixed dimer that can slide radially relative to the neighboring dimer, thus generating different helix tilt angles and pore sizes (Fig. 4f; **Extended Data movie 1**). Switching from one pore size to the other requires that all variable helix interfaces change. A fixed dimer can also slide longitudinally relative to its neighbor. This movement explains the two distinct variable interfaces in the mutant channel and results in only minimal changes of the pore size. Longitudinal sliding might explain the low resolution of the wild-type protein structures.

The partitioning of the receptor/cargo complex into the YG phase could shift the equilibrium from the resting state (~52 Å pore) to the active pores, possibly by expansion of the volume of the YG phase, which in turn could push the walls outwards. Oligomeric cargoes can have more than one bound receptor molecule, so their simultaneous partitioning into the YG phase could cause the conversion into the ~100 Å channel. Whether the channel adopts only discrete pore sizes (~52 Å, ~70 Å, ~100 Å) is unclear. Intermediates are conceivable, as the small side-chain amino acids make the variable interface promiscuous, probably allowing additional arrangements of the fixed dimers. Specifically, the change of the pore size from ~70 Å to ~100 Å is substantial, suggesting that intermediates may be required for this transition.

The iris-like changes of the channel pore are based on the unusual dual membrane topology of PEX13. This feature creates the constant and variable interfaces that enable helix sliding, and allows the YG phase to be suspended inside the membrane and be insulated from the surrounding lipid bilayer. It also places the SH3 domains of PEX13 alternately on either side of the membrane. SH3 domains facing the cytosol could recruit PEX5 to the channel by binding to the receptor’s aromatic motifs^41,42^. In yeast, SH3 domains facing the matrix could recruit PEX14^43^ but in mammals, PEX14 recruitment is mediated by different interactions^41^.

Our results support a new mechanism of protein translocation, distinct from previously described mechanisms that use either an aqueous channel or a locally thinned membrane to lower the energy barrier for moving a polypeptide across the membrane. An aqueous channel is formed by the prototypical Sec61 complex in the endoplasmic reticulum (ER) membrane and the analogous SecY complex in the plasma membrane of prokaryotes (for review, see^44^). Aqueous channels are also used for translocation across the outer membranes of mitochondria and chloroplasts^45,46^. In these cases, only unfolded polypeptides can move through the narrow channels. The locally thinned membrane mechanism is exemplified by bacterial YidC, which serves as an insertase of membrane proteins^47,48^, and the Hrd1 complex^49^, which is responsible for moving misfolded ER proteins into the cytosol for proteasomal degradation (ERAD). This mechanism is also used in other translocation systems, including that in the inner mitochondrial membrane (for review, see^50^). By contrast, our results suggest that the peroxisomal import channel has a unique cargo-adaptable pore that could allow folded and even oligomeric cargo to pass through^51^. The lining of the channel walls with amphipathic helices is also peculiar, found to our knowledge only in α-helical pore-forming toxins, whose pores are much smaller pores^52,53^.

The peroxisomal import channel resembles the nuclear pore. Both are formed from oligomeric assemblies of components with rotational symmetry, twelve in the case of PEX13 and multiples of eight in the case of nucleoporins. Like the peroxisomal channel, the nuclear pore contains a selective phase^16^ and can probably adjust to cargo size^37,38,54^. However, an important distinction is that the peroxisomal YG phase is positioned within the membrane, whereas the FG phase in nuclear pores is located in the aqueous space that connects cytosol and nucleoplasm. Nevertheless, the similarities between peroxisomal import and nuclear transport suggest common solutions to the challenge of transporting folded proteins between compartments.

## Supplementary Material

1

Materials and Methods

Extended Data Figs. 1 to 11

Extended Data Table 1

Extended Data Movie 1

References (55–62)

## Figures and Tables

**Fig. 1.**
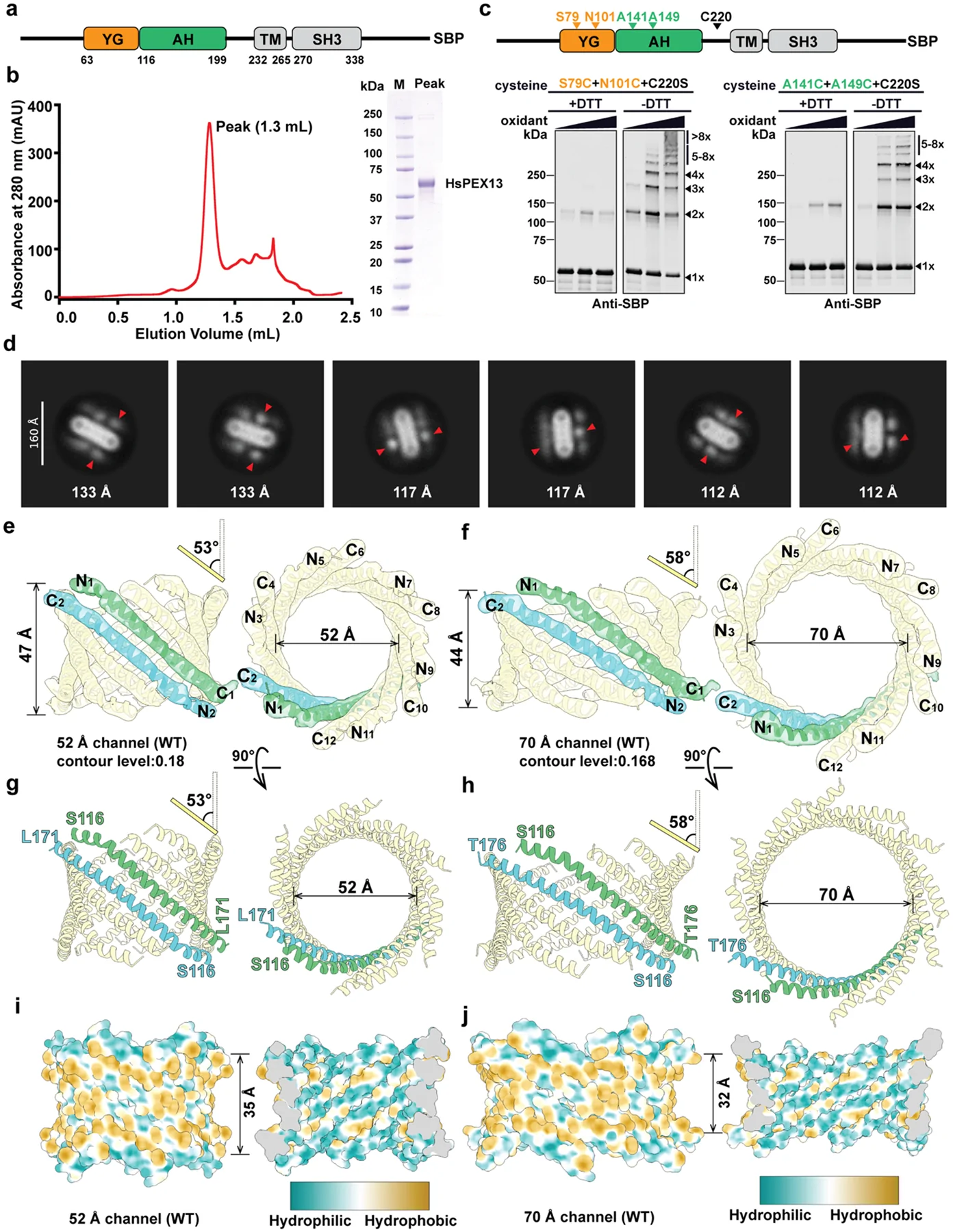
Cryo-EM structures of human PEX13. **a,** Domain organization of human PEX13. **b**, SBP-tagged PEX13 was expressed in *P. pastoris*. The protein was solubilized in digitonin and purified on streptavidin beads followed by SEC (left panel). The peak fraction was analyzed by SDS-PAGE and Coomassie blue staining (right panel). **c**, PEX13 containing two cysteines at the indicated positions of the YG or AH domains and with the endogenous cysteine 220 mutated to serine was purified as in (B) and treated with Cu-phenanthroline (oxidant). The samples were analyzed by nonreducing (–DTT) or reducing (+DTT) SDS-PAGE and immunoblotting with SBP antibodies. The number of crosslinked PEX13 molecules in the bands is indicated on the right. **d**, Purified human PEX13 was subjected to cryo-EM single-particle analysis. Shown are sideviews of 2D class averages. Arrowheads indicate SH3 domains on both sides of the detergent micelle. Note the variable lengths of the particles. **e**, Semi-transparent density map of the ~52 Å channel in two views, with a docked cartoon model based on AlphaFold3 (**Extended Data Fig. 4a**). Two of the 12 AHs are highlighted. The helix tilt angle is shown on top of the left panel. **f**, As in **e**, but for the ~70 Å channel. Another particle class has small C-terminal extensions of the AHs (**Extended Data Fig. 3a**). **g**, Model for the AHs of the ~ 52 Å channel. **h**, As in **g**, but for the ~70 Å channel. **i**, Space-filling model of the ~52 Å channel. The left panel shows the outside of the pore, and the right panel the inside in a cut-away view. Hydrophobic and hydrophilic residues are colored with the scale on the right. The size of the hydropobic belt is indicated. **j**, As in **i**, but for the ~70 Å channel.

**Fig. 2.**
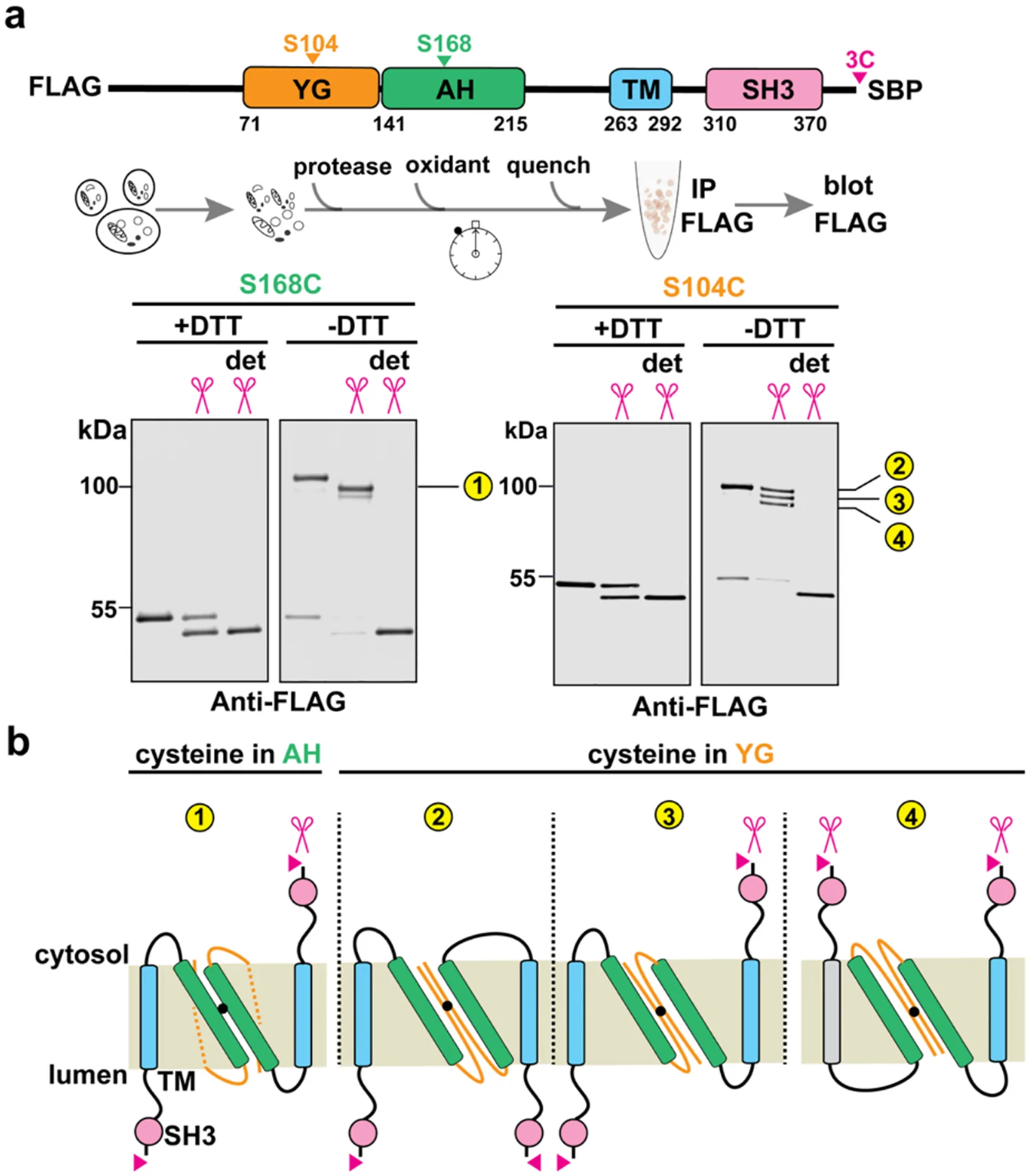
The AHs of *S. cerevisiae* PEX13 have alternating orientations. **a**, A cysteine was incorporated into the AH (green) or YG domains (orange) of FLAG-tagged yeast PEX13 containing a C-terminal 3C protease–cleavage site and SBP-tag, as indicated. The SBP tag serves to increase the size shift after proteolysis. Intact yeast membranes were treated first with the protease and then with the oxidant Cu-phenanthroline. The samples were quenched, and the proteins were immunoprecipitated (IP) and analyzed by nonreducing SDS-PAGE and immunoblotting with FLAG antibodies. Where indicated, dodecylmaltoside (det) was included during protease cleavage. The numbers refer to the topologies of the observed dimers (schemes in (**b**)). **b**, With the cysteine in the AH, only one crosslinked band is observed, containing AHs (in green) of opposite orientations. The topology of the downstream TM and SH3 domains is indicated. Red triangles show the 3C cleavage sites. Scissors show the sites accessible to added protease. With the cysteine in the YG domain, three bands are observed, corresponding to the three topologies shown (see also^14^).

**Fig. 3.**
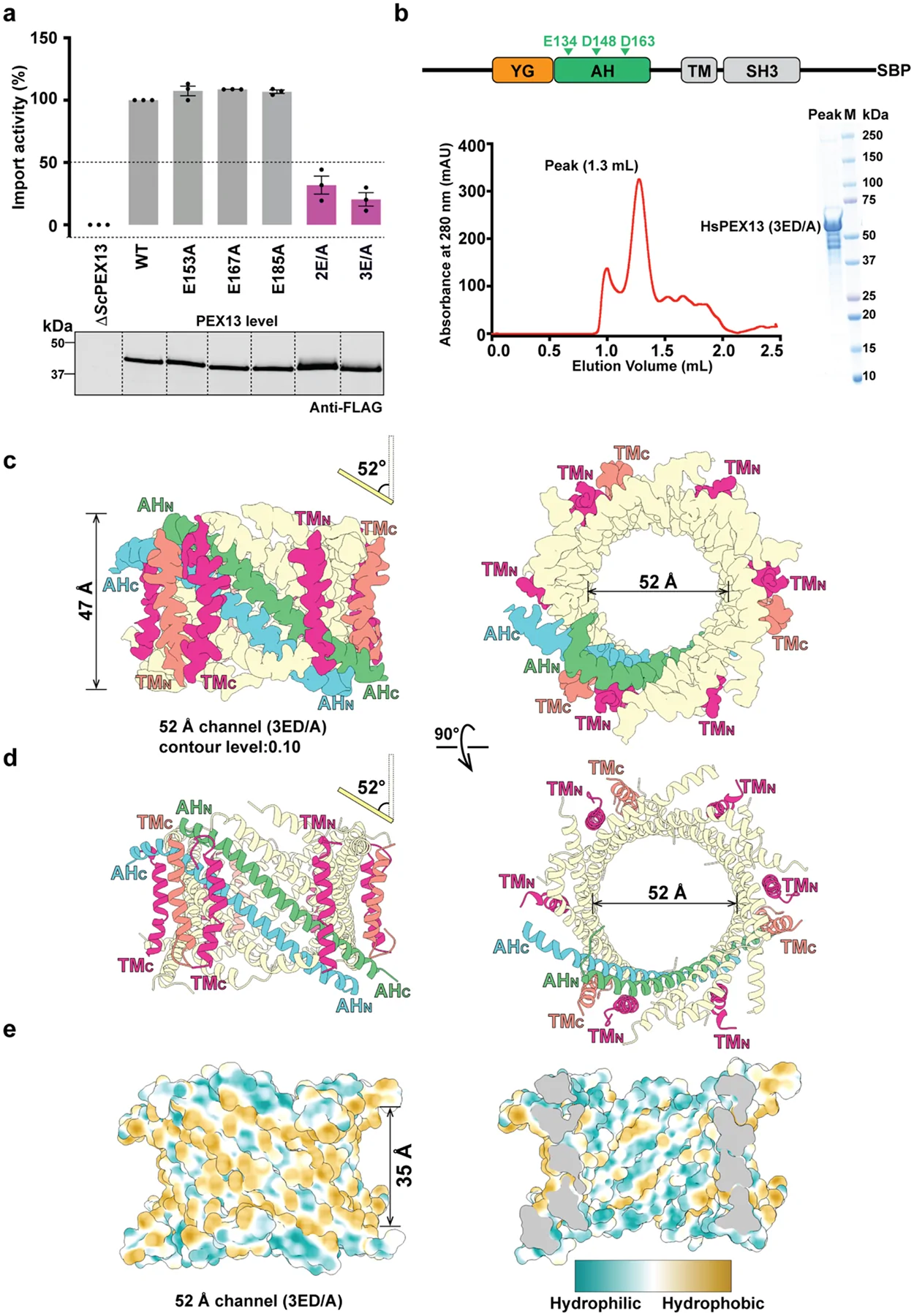
Cryo-EM structure of an inactive mutant channel. **a,** FLAG-tagged wild-type (WT) *S. cerevisiae* PEX13 or the indicated acidic residue mutants were expressed in *S. cerevisiae* cells lacking endogenous PEX13 (Δ*pex13*). Mutant 2E/A combines the E153A and E167A mutations, and 3E/A all three mutations. Peroxisomal protein import was measured with an engineered pathway that generates a pigment when import is impaired. Import activity is plotted relative to WT PEX13 (mean ± standard error of 3 experiments). The expression level of PEX13 is shown in the lower panel, analyzed by SDS-PAGE and immunoblotting with FLAG antibodies. **b**, Human PEX13 with three conserved acidic residues of the AH changed to alanines (3ED/A mutant) was expressed in *P. pastoris*. The protein was solubilized in digitonin, and purified with streptavidin beads followed by SEC (left panel). The peak fraction was analyzed by SDS-PAGE and Coomassie blue staining (right panel). **c**, Cryo-EM density map of the 3ED/A mutant channel in two views. Two AHs are highlighted in green and blue, with their N and C termini indicated. The helix tilt angle is shown on top of the left panel. Density for the TMs is colored salmon and red. **d**, Two views of the molecular model built into the map, shown as cartoons. **e**, Space-filling model showing the hydrophobic surface on the outside of the pore (left panel) and hydrophilic surface inside the pore (right panel). Hydrophobic and hydrophilic residues are colored with the scale on the right. The size of the hydrophobic belt is indicated.

**Fig. 4.**
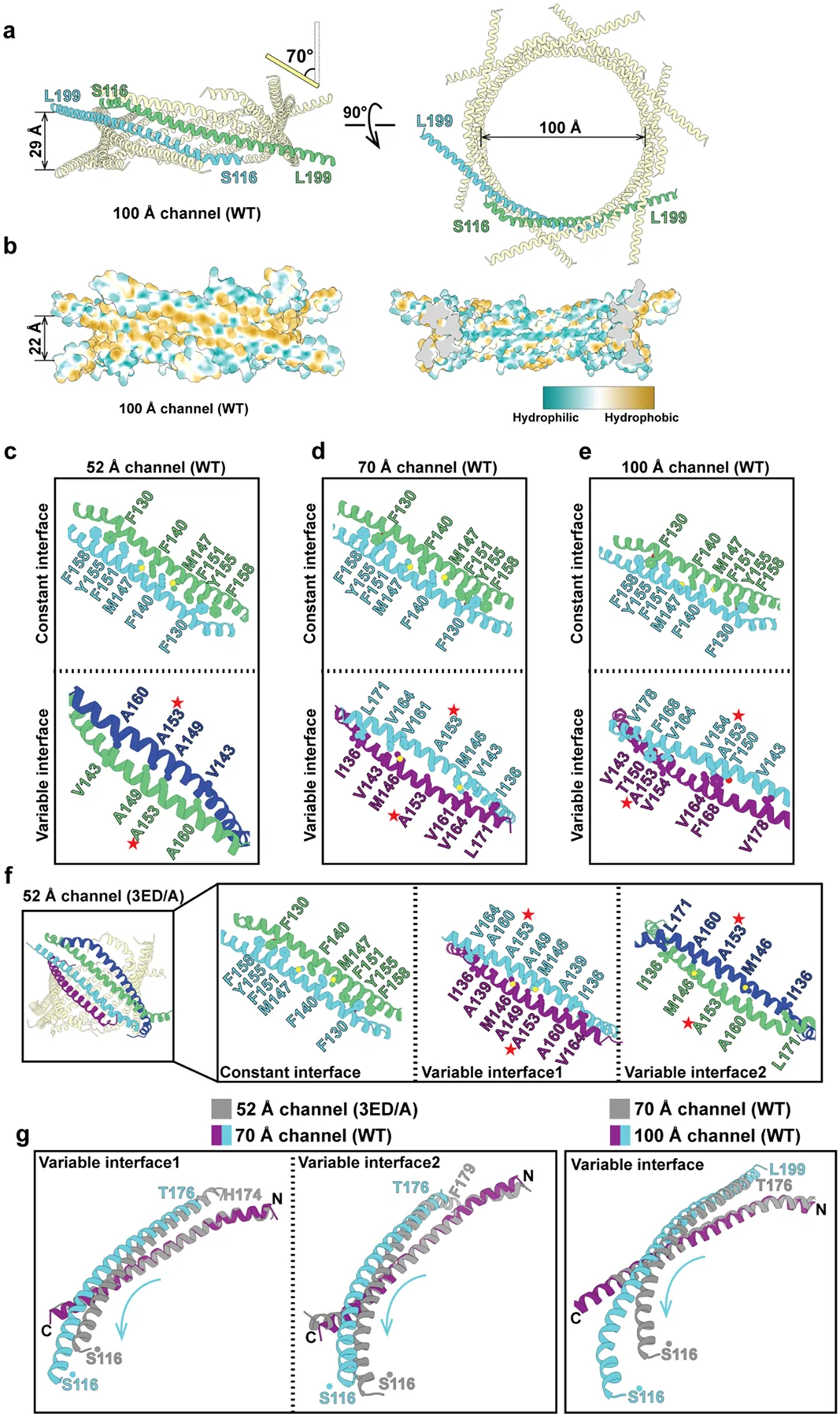
Helix sliding generates different pore sizes. **a**, Cartoon model of the AlphaFold2-predicted ~100 Å channel in two views. Two AHs are highlighted. Channel height and helix tilt angle are indicated. **b**, Space-filling model of the channel, showing the hydrophobicity of the outside and inside of the pore (scale on the bottom). The size of the hydropobic belt is indicated. **c**, Shown are the constant and variable interfaces of neighboring AHs in the ~52 Å wild-type (WT) channel. Interacting amino acids are labeled and shown with side chains. Hetero-atoms in side chains are shown in different colors. The position of the red star (residue 153) serves as a marker to show the relative movement of the helices. **d**, As in **c**, but for the ~70Å channel. **e**, As in **c**, but for the ~100 Å channel. **f**, As in **c**, but for the ~52 Å 3ED/A mutant channel. This channel two distinct variable interfaces (1 and 2) on either side of the fixed AH dimer. Interface 2 is similar to the variable interface of the ~52 Å wild-type channel. **f**, The left panels compare the variable interfaces of the ~52 Å 3ED/A mutant channel with that of the ~70 Å channel. One helix of the ~52 Å channel (grey) was aligned with a helix of the ~70 Å channel (purple). The movement of the other helix from the ~52 Å channel (grey) to the ~70 Å channel (blue) corresponds to an iris-like helix sliding. The right panel shows a comparison of the ~70 Å and ~100 Å channels.

**Fig. 5.**
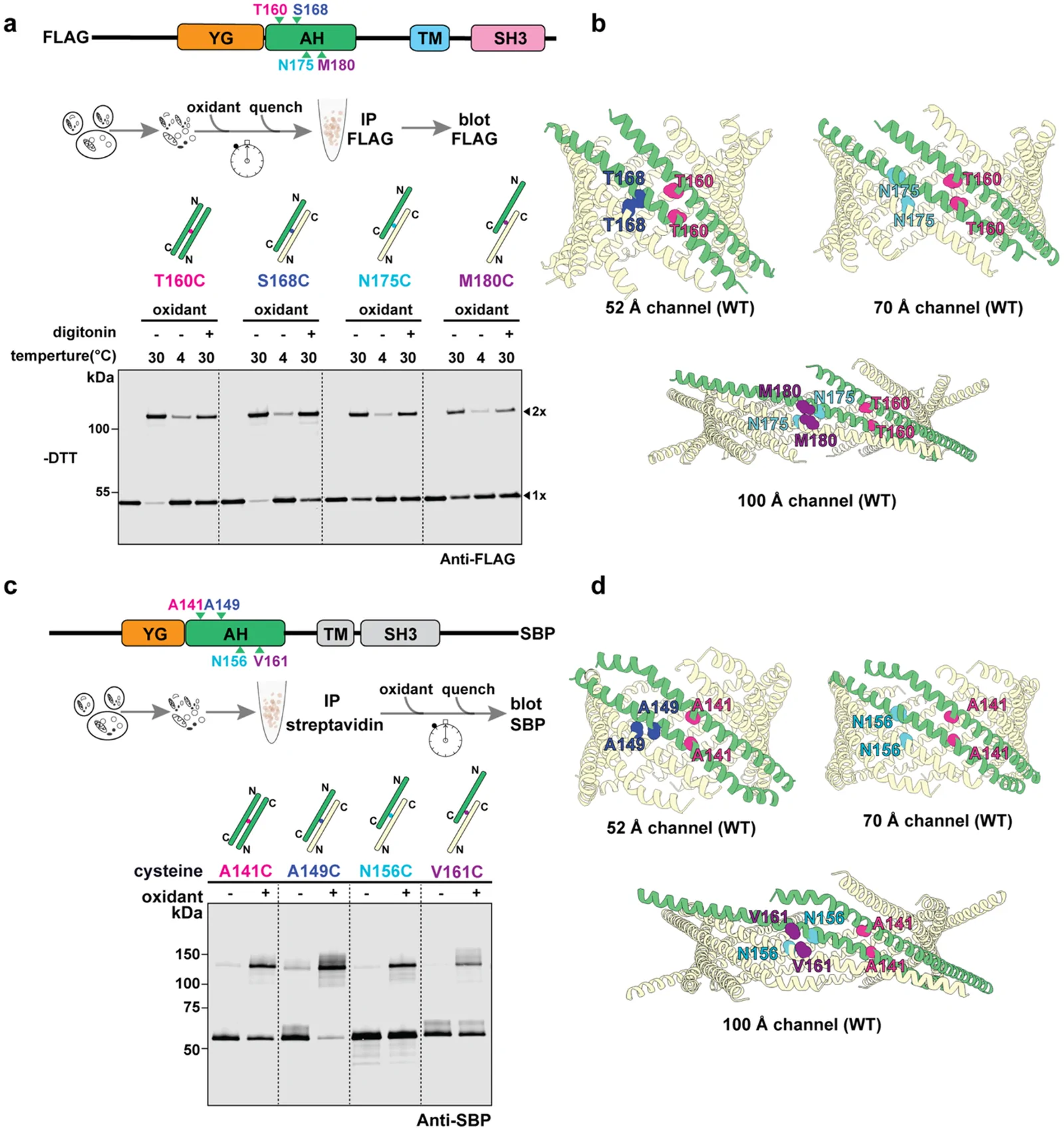
Different channel pores confirmed by disulfide crosslinking. **a**, FLAG-tagged PEX13 with cysteines at various positions of the AH was expressed in *S. cerevisiae*. Isolated membranes were treated with Cu-phenanthroline (oxidant) at 30°C or 4°C in the absence or presence of digitonin, as indicated. After quenching, the proteins were immunoprecipitated (IP) with FLAG antibodies and analyzed by nonreducing SDS-PAGE and immunoblotting with FLAG antibodies. Monomers (1x) and disulfide-linked dimers (2x) are indicated on the right. **b**, Cartoon models of the AlphaFold-predicted yeast channels. Two AHs connected by a constant interface are shown in green. Cysteines in neighboring AHs that explain disulfide-crosslinking in **a** are shown in the same color. Note that position 160 is in the constant interface; positions in the variable interface are diagnostic for the distinct channel states. **c**, SBP-tagged human PEX13 containing single cysteines at the indicated positions of the AH was expressed in *P. pastoris*. The proteins were solubilized in digitonin, purified with streptavidin beads, and treated with Cu-phenanthroline (oxidant) as indicated. The samples were analyzed by nonreducing SDS-PAGE and immunoblotting with SBP antibodies. **d**, As in **b**, but for the human channels.

**Fig. 6.**
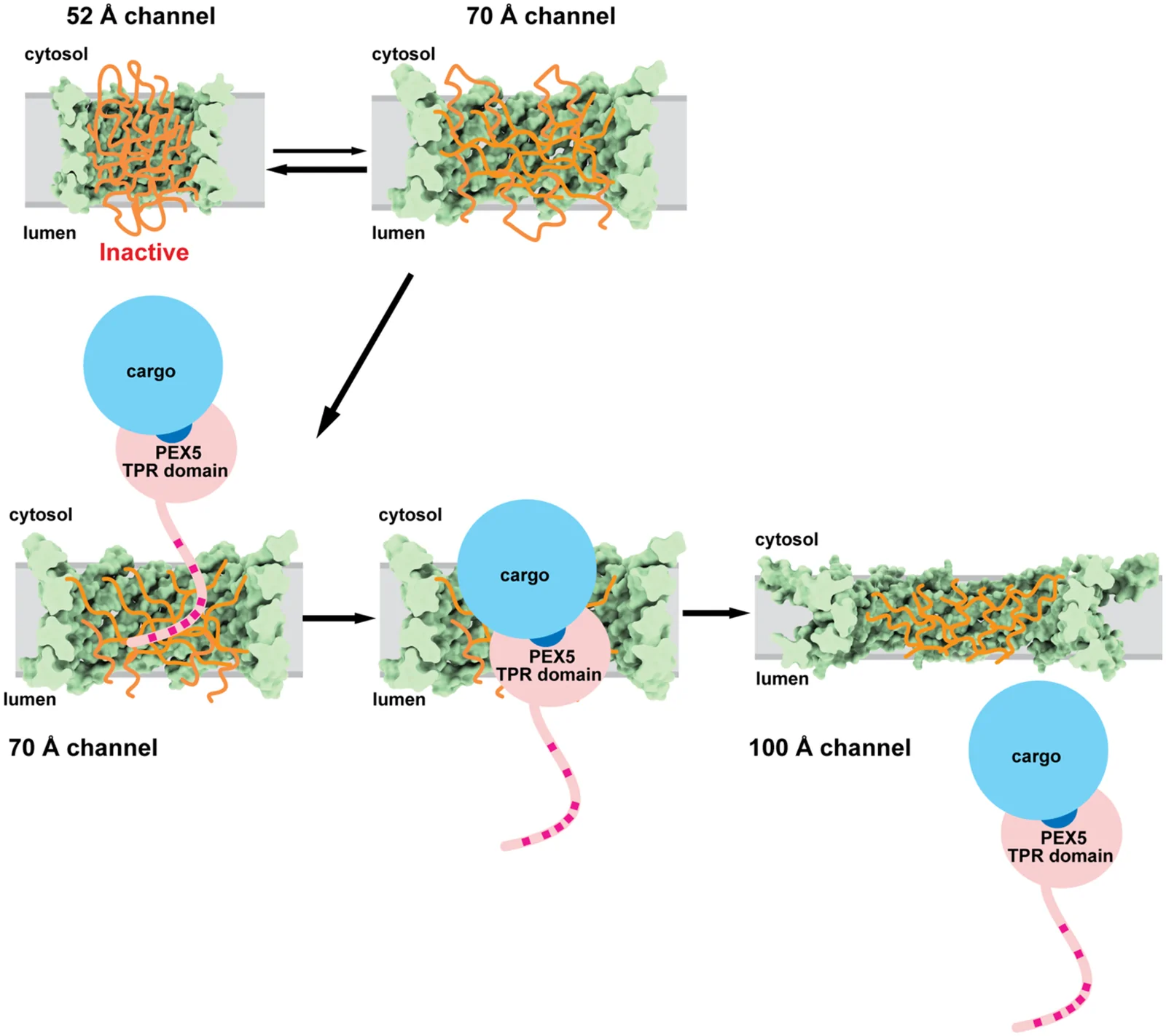
Protein import into peroxisomes reveals a novel translocation mechanism. Model of the cargo-size adaptable peroxisomal import channel. The different channels are shown as space-filling models in cut-away views. The YG meshwork is shown schematically in orange. The ~52 Å pore corresponds to an inactive resting state that is in equilibrium with the ~ 70 Å pore. The receptor PEX5 partitions into the YG phase through aromatic motifs in its unstructured N-terminus (red squares). Cargo containining a PTS1 sequence (dark blue) binds to the C-terminal TPR domain of PEX5. PTS2 cargo would additionally bind the adaptor PEX7 (not shown). The ~70 Å pore can widen to ~100 Å to allow peroxisomal import of large cargo.

## Data Availability

All data are available in the main text or the supplementary materials. Unique reagents generated by this study are available from the lead contact, Tom A. Rapoport (tom_rapoport@hms.harvard.edu) with a completed Materials Transfer Agreement. Cryo-EM maps and atomic coordinates generated in this work are available from the Electron Microscopy Data Bank (EMDB; EMD-78756, HsPEX13 52Å import channel (WT); EMD-78762, HsPEX13 70Å import channel (excluding 177–186); EMD-78763, HSPEX13 70Å import channel (including 177–186)). The accession codes for the HsPEX13 52Å import channel (3ED/A) are EMD-78822 in EMDB and PDB:38HQ in the Protein Data Bank.
