# Supplementary material for: An iris-like pore for protein import into peroxisomes
Source: bioRxiv. 2026 Oct 28:2026.10.23.761396. Preprint. [Version 2] doi: 10.64898/2026.10.23.761396 (PMC13733771; doi:10.64898/2026.10.23.761396)
Supplement: Supplementary file 1 [file NIHPP2026.10.23.761396V2-supplement-1.pdf]

bioRxiv preprint doi: https://doi.org/10.64898/2026.10.23.761396; this version posted October 28, 2026. The copyright holder for this preprint (which was not certified by peer review) is the author/funder, who has granted bioRxiv a license to display the preprint in perpetuity. It is made available under a CC-BY 4.0 International license.

Supplemental Material

Materials and Methods

All reagents were obtained from Millipore-Sigma unless specified otherwise. Buffers were prepared in ultrapure water. RT denotes room temperature.

Plasmids for expression of human PEX13 (HsPEX13) in *Pichia pastoris*

For overexpression of *HsPEX13* in *P. pastoris*, the DNA sequence encoding *HsPEX13* (UniProt accession no. Q92968) was synthesized and codon-optimized by Integrated DNA Technologies. *HsPEX13* carried at the N terminus three FLAG epitopes followed by a TEV protease cleavage site. The C terminus was fused to a streptavidin-binding protein (SBP) tag preceded by an HRV 3C protease cleavage site. All segments were assembled into the integrating vector pPICZα-LINK using Gibson assembly. For expression of the *HsPEX13*(3ED/A) mutant in *P. pastoris*, residues E134, D148, and D163 were mutated to alanines. For expression of the *HsPEX13* ΔYG (1–110) mutant, residues 1–110 of the YG domain were deleted. For disulfide crosslinking experiments, the residues indicated in the text were changed to cysteines. In these mutants, the endogenous cysteine residue at position 220 was mutated to serine (C220S). All deletions and single-residue mutants were generated by PCR or site-specific mutagenesis. For the HsPEX13 SG mutant, the tyrosine residues in the YG domain of *HsPEX13* (Y75, Y82, Y85, Y90, Y93, Y96, Y98, Y100, and Y105) were replaced with serines, cysteines were introduced at positions 79 and 101, and the endogenous cysteine at position 220 was changed to serine by *de novo* gene synthesis (Integrated DNA Technologies). All constructs were verified by whole-plasmid DNA sequencing.

Expression of HsPEX13 in *Pichia pastoris* and protein purification

P. pastoris wild-type strain SMD1168 was transformed by electroporation. Transformed yeast cells were grown on Zeocin-containing YPDS plates (YPD medium supplemented with sorbitol) at 30°C for 3–4 days. A single colony was picked to inoculate a starter culture, which was incubated overnight at 30°C. A large-scale culture was then inoculated by diluting the starter culture 1:80 into buffered minimal glycerol (BMG) medium. The culture was incubated at 30°C for approximately 24 h, after which protein expression was induced by transferring the cells into an equal volume of buffered minimal methanol (BMM)

bioRxiv preprint doi: https://doi.org/10.64898/2026.10.23.761396; this version posted October 28, 2026. The copyright holder for this preprint (which was not certified by peer review) is the author/funder, who has granted bioRxiv a license to display the preprint in perpetuity. It is made available under a CC-BY 4.0 International license.

medium. After an additional 18–20 h of incubation at 28 °C, the cells were harvested by centrifugation at 4,500 × *g* for 15 min at 4°C. The cell pellets were flash-frozen and stored at −80°C until further use.

For purification of wild-type *HsPEX13, the 3ED/A mutant, the* ΔYG mutant, and the SG mutant, ~10-15 g of cell pellets were resuspended in 80 ml of buffer A (400 mM NaCl and 25 mM HEPES, pH 7.5) supplemented with 1 mM phenylmethanesulfonyl fluoride (PMSF). Cells were lysed using a BioSpec BeadBeater for 50 min with 20 s on/60 s off cycles in a water–ice bath. The homogenate was centrifuged at 4,000 × *g* for 20 min to remove cell debris. The supernatant was then subjected to ultracentrifugation in a Ti45 rotor (Beckman Coulter) at 42,000 r.p.m. for 1 h at 4 °C. The membrane pellet was resuspended using a Dounce homogenizer in 20 ml of buffer A supplemented with 1% digitonin (EMD Millipore) and a protease inhibitor cocktail (Bimake, no. B14001), followed by incubation for 60 min at 4°C. Insoluble material was removed by centrifugation in a Ti70 rotor (Beckman Coulter) at 42,000 r.p.m. for 30 min. The supernatant was transferred to a new tube and incubated with 1 ml of high-capacity streptavidin resin (Thermo Scientific) for 1.5 h. The resin was washed with 20 ml of buffer A containing 0.1% digitonin, and the bound protein was eluted with 5 ml of buffer B (150 mM NaCl, 25 mM HEPES, pH 7.5, 10% glycerol and 0.1% digitonin) supplemented with 2 mM biotin (Sigma).The eluted protein was concentrated using a 100-kDa molecular-weight-cutoff filter (Millipore) and further purified by size-exclusion chromatography on a Superose 6 Increase 3.2/300 column (Cytiva Life Sciences), equilibrated with buffer C (25 mM HEPES, pH 7.5, 150 mM NaCl and 0.06% digitonin).

Disulfide-crosslinking of HsPEX13 variants

Single-cysteine mutant proteins of HsPEX13 were expressed in *P. pastoris* as above. The cells (0.1-0.5 g) were collected by centrifugation, washed once with water, and resuspended in ice-cold lysis buffer containing 300 mM NaCl and 25 mM HEPES (pH 7.5), supplemented with 1 mM PMSF and a protease inhibitor cocktail (Bimake, no. B14001). Each cell suspension was transferred to 2-ml screw-cap tubes (Sarstedt, no. 726994005) and kept on ice. Pre-chilled 0.5-mm glass beads were added, and the cells were lysed using a Mini-BeadBeater-16 (BioSpec Products, no. 607) in four 30-s cycles, with 2-min cooling intervals on ice in between. The lysate was clarified by centrifugation at 2,000 × g for 5 min at 4°C to remove intact cells and cell debris. The resulting supernatant was further centrifuged at 20,000 × g for 10 min at 4°C to pellet heavy membranes, including peroxisomes. The pellet was gently resuspended in lysis buffer supplemented with 1% digitonin by pipetting up and down, and then incubated on a rotator for 1 h at 4°C. Insoluble material was removed by centrifugation at 20,000 × g for 10 min at 4°C. The resulting supernatant was incubated with high-capacity streptavidin resin (Thermo Scientific) for 1 h at 4°C. The resin was washed with lysis buffer containing 0.1% digitonin, and the bound proteins were eluted with buffer B containing 150 mM NaCl, 25 mM HEPES (pH 7.5), 10% glycerol, and 0.1% digitonin, supplemented with 2 mM biotin. The eluted proteins were immediately snap-frozen and stored at −80°C until further use.

bioRxiv preprint doi: https://doi.org/10.64898/2026.10.23.761396; this version posted October 28, 2026. The copyright holder for this preprint (which was not certified by peer review) is the author/funder, who has granted bioRxiv a license to display the preprint in perpetuity. It is made available under a CC-BY 4.0 International license.

The samples were thawed and added to assay buffer containing 0.1% digitonin, 20 mM HEPES–NaOH (pH 7.5), 50 mM NaCl, 250 mM sucrose, and 1 mM EDTA, and then treated with the oxidizing agent copper(II)-phenanthroline at 0, 0.3 mM, or 0.5 mM for cysteines in the AH, and at 0, 0.05 mM, or 0.2 mM for cysteines in the YG domain. The samples were incubated for 30 min at 30°C with gentle agitation, quenched with 10 mM N-ethylmaleimide (NEM). One half of the sample received 100 mM dithiothreitol (DTT) and both were heated at 95ºC in Laemmli buffer for SDS-PAGE and immunoblotting.

For disulfide crosslinking with two cysteines incorporated into HsPEX13, the following mutants were used: S79C, N101C, C220S; A141C, A149C, C220S; A141C, N156C,C220S; A141C, V161C,C220S; and A141C, A149C, C220S, E134A, D148A, D163A. These proteins were purified as above, except that they were further purified by SEC on a Superose 6 Increase column. The peak fractions were used for disulfide crosslinking as described before.

Peroxisomal import assay in *S. cerevisiae* cells

A modified violacein biosynthetic pathway [27] was used to quantitatively measure peroxisomal matrix protein import in *S. cerevisiae* cells, as described [11]. In this system, the first two enzymes of the pathway (VioA and VioB) reside in the cytosol, whereas the third enzyme (VioE) contains a peroxisome targeting signal and is sequestered inside peroxisomes when import is functional. Only when import is compromised does VioE accumulate in the cytosol and the green pigment is made. Pigment production is inversely proportional to import efficiency [27].

Import was measured in a strain (UTL7A (*MATa, ura3-52, trp1, leu2-3/112*)) that contains an expression cassette encoding the three enzymes (VioA, VioB, and VioE) integrated into the genome along with a leucine auxotrophic marker. The *pex13* gene was deleted and replaced with the *natMX* marker (nourseothricin resistance), as described [11]. This strain was transformed with PCR-generated DNA fragments coding either for wild-type PEX13 or for PEX13 mutants carrying changes of the negatively charged residues (E153, E167, E185) to alainines, all with 60-bp homology overhangs. The PCR products were introduced into yeast cells by lithium acetate–based transformation [48]. Transformants were selected on YPD medium containing hygromycin (250 µg/ml; Fisher scientific). Correct integration of the mutant or epitope-tagged coding sequence was validated by PCR and DNA sequencing.

For pigment quantification, yeast strains were cultured overnight in YPD medium at 30°C with shaking. The next morning, cultures were diluted 150-fold into 3 ml of freshly prepared synthetic defined (SD) medium lacking leucine and incubated at 30°C with shaking for an additional 50 h. Cells were collected by centrifugation and resuspended in 300 µl of glacial acetic acid (Fisher Scientific). The cell suspensions were transferred to 1.5-ml microcentrifuge tubes, incubated at 95°C for 10 min, mixed by inversion, and incubated for an additional 10 min at 95°C. Cell debris were removed by centrifugation at 13,000 × *g* for 5 min at room temperature. Subsequently, 200 µl of each supernatant was transferred to a 96-well, round-bottom, black-walled plate (Corning, no. 3792). Fluorescence was measured using a BioTek Synergy Neo2 microplate reader with excitation and emission wavelengths of 535 ± 10 nm and 585 ± 10 nm, respectively. Relative peroxisomal matrix protein import activity was calculated by normalizing the fluorescence signal

bioRxiv preprint doi: https://doi.org/10.64898/2026.10.23.761396; this version posted October 28, 2026. The copyright holder for this preprint (which was not certified by peer review) is the author/funder, who has granted bioRxiv a license to display the preprint in perpetuity. It is made available under a CC-BY 4.0 International license.

of each strain to the average fluorescence of wild-type cells (set to 100%) and *pex13Δ* cells (set to 0%), using linear interpolation.

To assess the expression levels of the FLAG-tagged PEX13 constructs, membranes from the relevant strains were solubilized in 1.0% (w/v) dodecylmaltoside (DDM), and subjected to immunoprecipitation using anti-FLAG M2 beads before analysis by SDS-PAGE.

To evaluate the functional significance of the constant and variable AH interfaces, Y170, H174, and F177 at the constant interface were mutated to alanines, and A172 at the variable interface was mutated to phenylalanine. All constructs encoding the PEX13 variants were transformed into yeast cells, and peroxisomal import activity was measured as described before. To assess protein expression levels, all PEX13 constructs carried a FLAG tag. Expression levels were determined by SDS-PAGE followed by immunoblotting with FLAG antibodies.

Disulfide-crosslinking of *S. cerevisiae* PEX13

The *Δpex13* strain described above was transformed with PCR products coding for FLAG-tagged PEX13 variants containing the indicated cysteine substitutions to integrate the genes into the genome. The cells were cultured overnight in YPD medium at 30°C, collected by centrifugation, washed once with water, and resuspended in ice-cold lysis buffer containing 20 mM HEPES–KOH (pH 6.8 at room temperature), 150 mM potassium acetate, 5 mM magnesium acetate, 250 mM sorbitol, and 1 mM EDTA, supplemented with 1 mM PMSF and a protease inhibitor cocktail (Bimake, no. B14001). The cell suspension was transferred to 2-ml screw-cap tubes (Sarstedt, no. 726994005) and kept on ice. Prechilled 0.5-mm glass beads were added, and cells were lysed using a Mini-BeadBeater-16 (BioSpec Products) for four 30-s cycles, with 2-min cooling intervals on ice between cycles. The lysate was clarified by centrifugation at 2,000 × g for 5 min at 4°C to remove intact cells and cell debris. The resulting supernatant was centrifuged at 20,000 × g for 10 min at 4°C to pellet peroxisomes. The final pellet was gently resuspended in assay buffer containing 20 mM HEPES–NaOH (pH 7.2), 50 mM NaCl, 250 mM sucrose, and 1 mM EDTA, followed by incubation on a rotator at 4°C for 15 min.

The homogeneous membrane suspension was treated with or without the oxidizing agent copper (II)-phenanthroline (Cu-phenanthroline) at 0.1 mM for 30 min at 30°C or 4°C with gentle agitation. Where indicated, 1.0 % digitonin was added before the oxidant to solubilize the membranes. Unreacted cysteine residues were quenched with 10 mM NEM at 30°C for 5 min. PEX13 was immunoprecipitated from the resulting supernatant using anti-FLAG M2 agarose beads (Millipore, no. A2220) and analyzed by SDS-PAGE.

Determination of the membrane topology of the AH in *S. cerevisiae*

A single cysteine was introduced at position 168 (AH) into PEX13 containing a FLAG tag at its N-terminus and a 3C protease-cleavage site and SBP tag at the C terminus. The construct was integrated into the chromosome. An analogous strain with a cysteine at position 104 (YG domain) has been described before [11]. Membranes from these strains were prepared as

bioRxiv preprint doi: https://doi.org/10.64898/2026.10.23.761396; this version posted October 28, 2026. The copyright holder for this preprint (which was not certified by peer review) is the author/funder, who has granted bioRxiv a license to display the preprint in perpetuity. It is made available under a CC-BY 4.0 International license.

described above, mixed with 2 μM homemade 3C protease, and incubated for 16 h at 4°C. The samples were then treated with copper(II)-phenanthroline (0.1 mM) for 30 min at 30°C to promote disulfide bond formation. The reactions were subsequently quenched with 10 mM NEM and the membranes were solubilized with 0.5% dodecylmaltoside (DDM) for 1 h at 4°C with shaking. PEX13 was then immunoprecipitated from the resulting supernatant using anti-FLAG M2 agarose. The precipitated material was eluted with 0.4 mg/mL 3×FLAG peptide (Bimake, no. B23112) in assay buffer containing 0.05% (w/v) DDM and prepared for reducing or non-reducing SDS-PAGE.

Electrophoresis and immunoblotting

Samples were analyzed by SDS- PAGE on 4–20% TGX precast polyacrylamide gels (Bio-Rad). For immunoblotting, proteins were transferred onto nitrocellulose membranes (Bio-Rad, no. 1620112) using Trans-Blot turbo Transfer System (BioRad). Membranes were blocked in TBST buffer (20 mM Tris–NaOH, pH 7.5, 150 mM NaCl, and 0.1% (v/v) Tween 20) containing 3% (w/v) non-fat milk solids (Apex, no. 20241) and then incubated with antibodies against the FLAG epitope (Millipore Sigma, no. F7425) or SBP tag (Millipore Sigma, no. MAB10764), followed by fluorescently labeled secondary antibodies IRDye 800CW (LI-COR Biosciences), as appropriate. Blots were imaged using a LI-COR Odyssey M imaging system.

Cryo-EM sample preparation and data acquisition

For cryo-EM sample preparation of wild-type *HsPEX13,* the peak fractions obtained from SEC were concentrated to ~2.6 mg/ml. Three μl of the sample were applied to Quantifoil Cu 1.2/1.3 400 mesh grids that were glow-discharged at 15 mA for 30 s using a PELCO glow-discharge system (Ted Pella). The grids were blotted for 7.0 s at a blot force of 20 and ~100% humidity and then plunge-frozen in liquid ethane using a Vitrobot Mark IV instrument (Thermo Fisher Scientific). For cryo-EM sample preparation of *HsPEX13* (3ED/A), the SEC peak fractions were concentrated to ~5.0 mg/ml. Three μl of the sample were applied to a glow-discharged Quantifoil grid (1.2/1.3, Au, 300 mesh). The grids were blotted for 6.0 s at a blot force of 6 and ~100% humidity and then plunge-frozen in liquid ethane using a Vitrobot Mark IV instrument. All grids were stored in liquid N_2.

Cryo-EM data were collected on a Titan Krios electron microscope (FEI) operated at 300 kV and equipped with a post-GIF Gatan Summit direct electron detector K3 camera (Gatan) at the HHMI Janelia Research Campus Cryo-EM Facility. For wild-type *HsPEX13,* images were collected on Krios2. An energy-filter slit width of 20 eV was used to remove inelastically scattered electrons. All cryo-EM movies were recorded in super-resolution counting mode using SerialEM. A nominal magnification of ×81,000 corresponded to a calibrated physical pixel size of 1.06 Å per pixel and a super-resolution pixel size of 0.53 Å per pixel. The total electron dose was 50 electrons Å^{-2}, fractionated over 50 frames. The defocus range was set from −1.0 to −2.5 μm. A total of 9,697 movies were recorded over 3 three days.

bioRxiv preprint doi: https://doi.org/10.64898/2026.10.23.761396; this version posted October 28, 2026. The copyright holder for this preprint (which was not certified by peer review) is the author/funder, who has granted bioRxiv a license to display the preprint in perpetuity. It is made available under a CC-BY 4.0 International license.

For the *HsPEX13* (3ED/A), images were collected on Krios1. All cryo-EM movies were recorded in super-resolution counting mode using SerialEM. A nominal magnification of ×81,000 corresponded to a calibrated physical pixel size of 0.857 Å per pixel and a super-resolution pixel size of 0.4285 Å per pixel. The defocus range was set from −1.0 to −1.8 µm. A total of 17,301 movies were recorded over 3 days. All other data-collection parameters were the same as those used for the wild-type dataset.

Data processing

For wild-type (WT) HsPEX13, cryo-EM data were processed as summarized in **fig. S2**. Data processing was performed using cryoSPARC v4.7.1 [49]. Following patch motion correction, patch-based contrast transfer function (CTF) estimation, and manual inspection to remove micrographs with ice contamination or poor image quality, particles were picked from a random subset of 500 micrographs using the cryoSPARC Blob Picker. Two-dimensional (2D) classification was then performed to generate 2D templates, and particles were autopicked from 8,712 micrographs using these templates with the cryoSPARC Template Picker and Inspect Particles, yielding a total of 1,561,361 particles. Particles were initially extracted into 180 × 180-pixel boxes with 2× downscaling and subjected to two rounds of reference-free 2D classification. Poorly populated 2D classes were removed, yielding a selected set of 650,685 particles. These particles were then re-extracted into 360 × 360-pixel boxes and subjected to *ab initio* reconstruction (N=6) to generate initial 3D models. *Ab initio* reconstruction was performed with a maximum resolution of 6 Å, an initial resolution of 9 Å, an initial minibatch size of 300, a final minibatch size of 1,000, and C6 symmetry. Two distinct reconstruction classes were obtained, corresponding to large (~70 Å channel)- and small (~52 Å channel)- size particles, containing 172,462 and 135,666 particles, respectively.

For the ~70 Å channel reconstruction, two distinct classes were obtained after two rounds of ab-initio reconstruction (N=3). One reconstruction lacked density corresponding to the tail region (residues K177–L186) and contained 50,745 particles, whereas the other contained 49,051 particles. Each particle subset was subjected to non-uniform refinement [50] with C6 symmetry, yielding global resolutions of 6.18 Å and 6.23 Å, respectively.

For the small-size reconstruction, 36,627 particles from the cleanest 3D class were selected after one round of ab-initio reconstruction (N=5) and subjected to non-uniform refinement with C6 symmetry, yielding a global resolution of 6.2 Å. Otherwise, the parameters used for ab initio reconstruction were the same as above.

The data processing for the HsPEX13 (3ED/A) dataset is summarized in **Extended Data Fig. 2**. Data processing was performed using cryoSPARC v4.7.0 [49], as described above, with particles picked from a random subset of 3,517 micrographs by cryoSPARC Blob Picker. Two-

bioRxiv preprint doi: https://doi.org/10.64898/2026.10.23.761396; this version posted October 28, 2026. The copyright holder for this preprint (which was not certified by peer review) is the author/funder, who has granted bioRxiv a license to display the preprint in perpetuity. It is made available under a CC-BY 4.0 International license.

dimensional (2D) classification was then performed to generate 2D templates, and particles were autopicked from 15,130 micrographs using these templates with the cryoSPARC Template Picker and Inspect Particles, yielding a total of 3,213,428 particles. Particles were initially extracted into 180 × 180-pixel boxes and subjected to two rounds of reference-free 2D classification. Poorly populated 2D classes were removed, yielding a selected set of 704,254 particles. These particles were then re-extracted into 360 × 360-pixel boxes and subjected to an additional round of 2D classification to yield 586,960 particles. The resulting particle set was subjected to two rounds of ab-initio reconstruction (N = 6 in the first round and N = 3 in the second round) to generate initial 3D models. Ab-initio reconstruction was performed with a maximum resolution of 6 Å, an initial resolution of 9 Å, an initial minibatch size of 300, a final minibatch size of 1,000, and C3 symmetry. One reconstruction class was selected, containing 102,845 particles. This particle set was subsequently subjected to one round of heterogeneous refinement (N=3) using a 360-pixel refinement box and C3 symmetry, yielding a final particle set of 75,550 particles. The resulting particle set was subjected to non-uniform refinement with C1 or C3 symmetry, resulting in global resolutions of 3.3 Å and 2.8 Å repectively. Histograms of directional Fourier shell correlation curves and sphericity values were calculated with the 3DFSC server [51]. All software used in this study was supported by SBGrid [52].

Model building

Wild-type HsPEX13 models were derived from AlphaFold-predicted models. For the 52 Å model, the amphipathic helix (S116–L171) was fitted into the small-size density map using ChimeraX 1.8 [53]. TM helices and portions of the AH that extended beyond the density were removed in Coot [54].

For the ~70 Å structure without the tail region, the AH (S116–T176) was fitted into the density map, and portions of the model that extended beyond the density were trimmed in Coot. For the 70 Å model containing the tail region (K177–R186), the AlphaFold model was first fitted into the density map, followed by manual adjustment of residues K177–R186 to better fit the corresponding density. Additional regions of the AlphaFold model that were not supported by the density were removed.

For the 52 Å channel of *HsPEX13* (3ED/A), the small-size AlphaFold model was docked into the density map and subsequently transferred to Coot for manual model building. The model was refined through iterative rounds of automated refinement in Phenix [55]. Local resolution

bioRxiv preprint doi: https://doi.org/10.64898/2026.10.23.761396; this version posted October 28, 2026. The copyright holder for this preprint (which was not certified by peer review) is the author/funder, who has granted bioRxiv a license to display the preprint in perpetuity. It is made available under a CC-BY 4.0 International license.

was estimated using cryoSPARC [49]. Structural visualization and analysis were performed using ChimeraX 1.8 [53].

To model PEX5 inside the ~70 Å channel, the TPR domain of HsPEX5 (PDB: 9GAG) was manually docked into the channel. Modeling of receptor–cargo complexes inside the channel was done with ScPEX5 TPR domain associated with Eci1 (PDB: 9FH0) and with HsPEX5 TPR domain associated with mSCP2 (PDB: 2C0L). Docking was performed with ChimeraX 1.8.

AlphaFold predictions of PEX13 structures

The ~52 Å, ~80 Å and ~70 Å channel models of *HsPEX13* and *Sc*PEX13 were predicted using AlphaFold3 through the AlphaFold Server (https://alphafoldserver.com/) The ~100 Å channel models of *HsPEX13* and *Sc*PEX13 were predicted using AlphaFold2 ColabFold through the COSMIC2 server (https://www.cosmic2.org/).

In initial predictions, we used 12 copies of the AH of HsPEX13, spanning residues S116–G200, and identified channel states with approximately 52 Å ,70 Å, and 80 Å in diameter. However, the prediction confidence scores were low. We found significant improvement of the predictions when *12* copies of AHs, comprising residues S116–G200, were supplemented with 12 copies of the TM, comprising residues A227–T256. In this case, only channels of ~52Å, and ~70Å diameter were predicted. For the ~100 Å channel, 12 copies of the AHs, comprising residues S116–L199, were used without inclusion of the TM.

For the ~52 Å and ~70 Å channels of *Sc*PEX13, 12 copies of the residues L141–G299, including both the AH and TM regions, were used for the predictions. For the ~100 Å channel, 12 copies of residues S139–N250, with the TM excluded, were used.

bioRxiv preprint doi: https://doi.org/10.64898/2026.10.23.761396; this version posted October 28, 2026. The copyright holder for this preprint (which was not certified by peer review) is the author/funder, who has granted bioRxiv a license to display the preprint in perpetuity. It is made available under a CC-BY 4.0 International license.

References

55 Gietz, R. D. & Schiestl, R. H. High-efficiency yeast transformation using the LiAc/SS carrier DNA/PEG method. *Nat Protoc* **2**, 31-34, doi:10.1038/nprot.2007.13 (2007).

56 Punjani, A., Rubinstein, J. L., Fleet, D. J. & Brubaker, M. A. cryoSPARC: algorithms for rapid unsupervised cryo-EM structure determination. *Nat Methods* **14**, 290-296, doi:10.1038/nmeth.4169 (2017).

57 Punjani, A., Zhang, H. & Fleet, D. J. Non-uniform refinement: adaptive regularization improves single-particle cryo-EM reconstruction. *Nat Methods* **17**, 1214-1221, doi:10.1038/s41592-020-00990-8 (2020).

58 Tan, Y. Z. *et al.* Addressing preferred specimen orientation in single-particle cryo-EM through tilting. *Nat Methods* **14**, 793-796, doi:10.1038/nmeth.4347 (2017).

59 Morin, A. *et al.* Collaboration gets the most out of software. *Elife* **2**, e01456, doi:10.7554/eLife.01456 (2013).

60 Pettersen, E. F. *et al.* UCSF ChimeraX: Structure visualization for researchers, educators, and developers. *Protein Sci* **30**, 70-82, doi:10.1002/pro.3943 (2021).

61 Emsley, P. & Cowtan, K. Coot: model-building tools for molecular graphics. *Acta Crystallogr D Biol Crystallogr* **60**, 2126-2132, doi:10.1107/S0907444904019158 (2004).

62 Afonine, P. V. *et al.* Real-space refinement in PHENIX for cryo-EM and crystallography. *Acta Crystallogr D Struct Biol* **74**, 531-544, doi:10.1107/S2059798318006551 (2018).

bioRxiv preprint doi: https://doi.org/10.64898/2026.10.23.761396; this version posted October 28, 2026. The copyright holder for this preprint (which was not certified by peer review) is the author/funder, who has granted bioRxiv a license to display the preprint in perpetuity. It is made available under a CC-BY 4.0 International license.

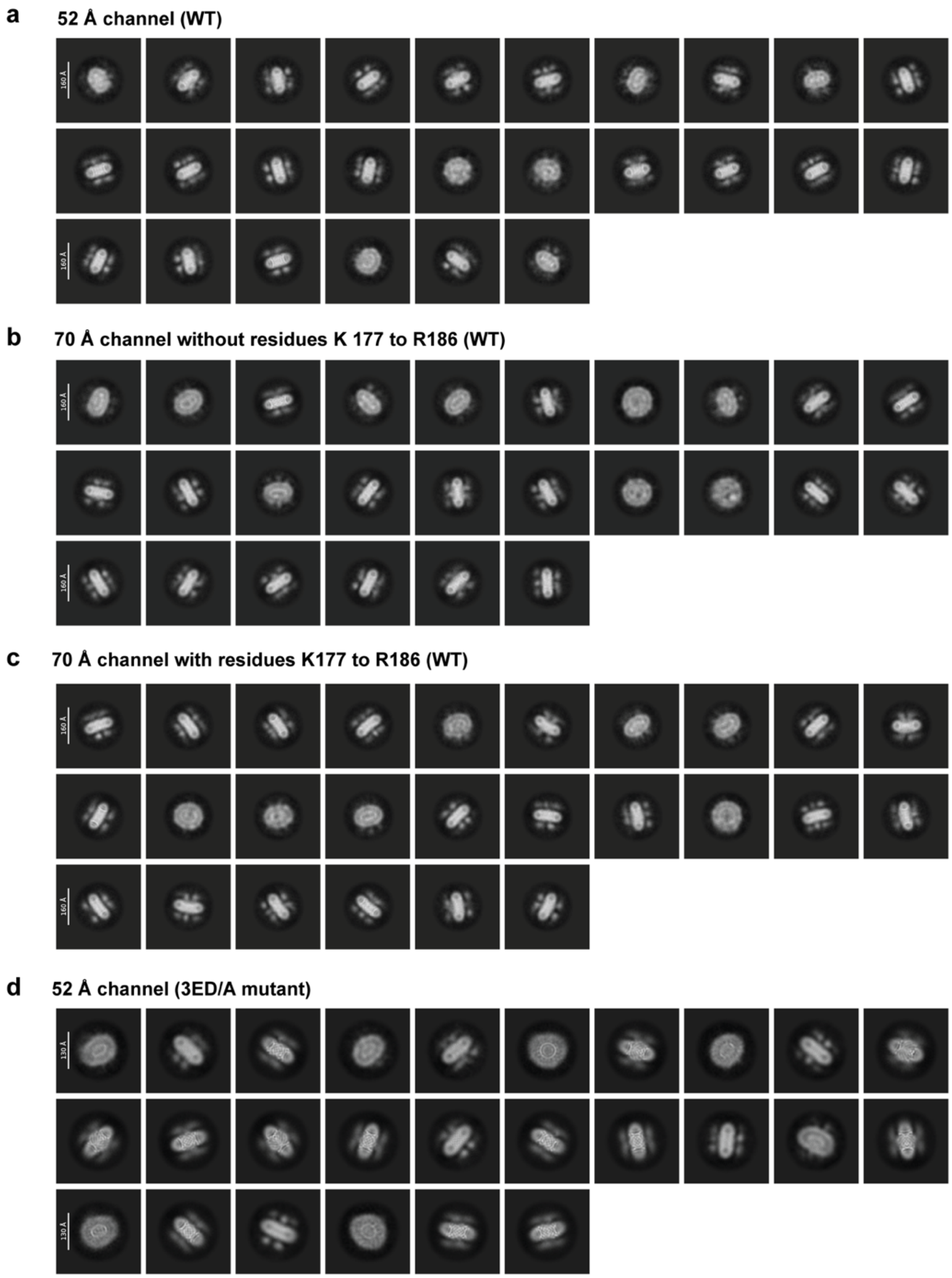

Extended Data Fig. 1 | Cryo-EM 2D average classes of different channels.

a, 2D average classes obtained for the ~52 Å wild-type (WT) channel. **b**, As in **a**, but for the ~70 Å wild-type channel lacking density for residues 177-186. **c**, As in **a**, but for the ~70 Å wild-type channel containing density for residues 177-186. **d**, As in **a**, but for the 3ED/A mutant.

bioRxiv preprint doi: https://doi.org/10.64898/2026.10.23.761396; this version posted October 28, 2026. The copyright holder for this preprint (which was not certified by peer review) is the author/funder, who has granted bioRxiv a license to display the preprint in perpetuity. It is made available under a CC-BY 4.0 International license.

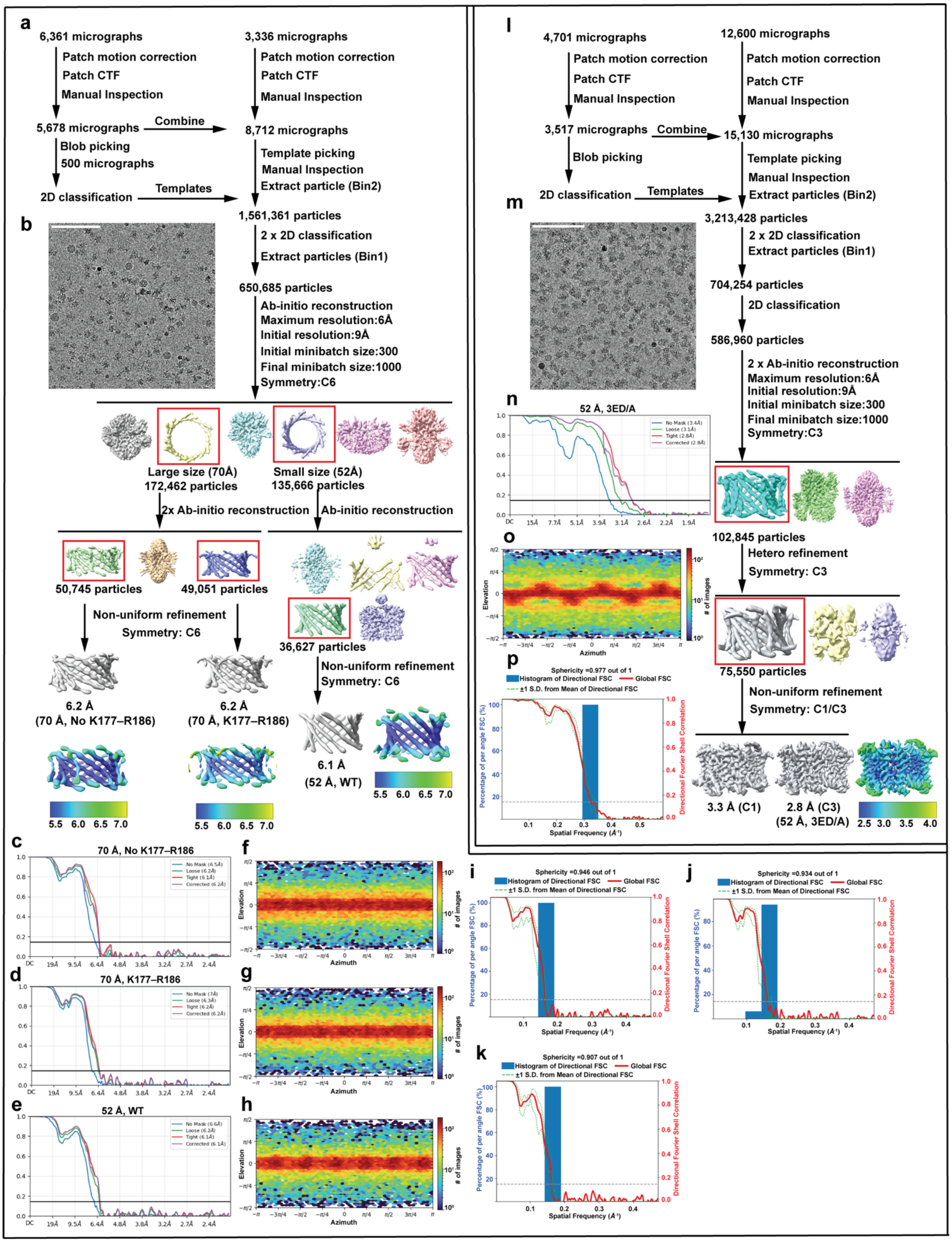

bioRxiv preprint doi: https://doi.org/10.64898/2026.10.23.761396; this version posted October 28, 2026. The copyright holder for this preprint (which was not certified by peer review) is the author/funder, who has granted bioRxiv a license to display the preprint in perpetuity. It is made available under a CC-BY 4.0 International license.

Extended Data Fig. 2 | Cryo-EM data processing.

a, Data-processing scheme for wild-type human PEX13. The particles classes boxed in red were further processed, as indicated. In the final reconstruction, the density map is colored according to local resolution. **b**, Representative raw micrograph. **c**-**e**, Fourier shell correlation (FSC) plots for the ~70 Å channel map lacking the segment K177–R186, for the ~70 Å channel map containing the K177–R186 segment, and for the ~52 Å channel map, respectively. **f**-**h**, Particle orientation distributions for the three wild-type PEX13 maps. **i**-**k**, 3D FSC plots for the three maps. **l**, As in **a**, but for the 3ED/A mutant protein. **m**, As in **b**, but for the 3ED/A mutant protein. **n**, As in **c**-**e**, but for the 3ED/A mutant protein. **o**, As in **f**-**h**, but for the 3ED/A mutant protein. **p**, As in **i**-**k** , but for the 3ED/A mutant protein.

bioRxiv preprint doi: https://doi.org/10.64898/2026.10.23.761396; this version posted October 28, 2026. The copyright holder for this preprint (which was not certified by peer review) is the author/funder, who has granted bioRxiv a license to display the preprint in perpetuity. It is made available under a CC-BY 4.0 International license.

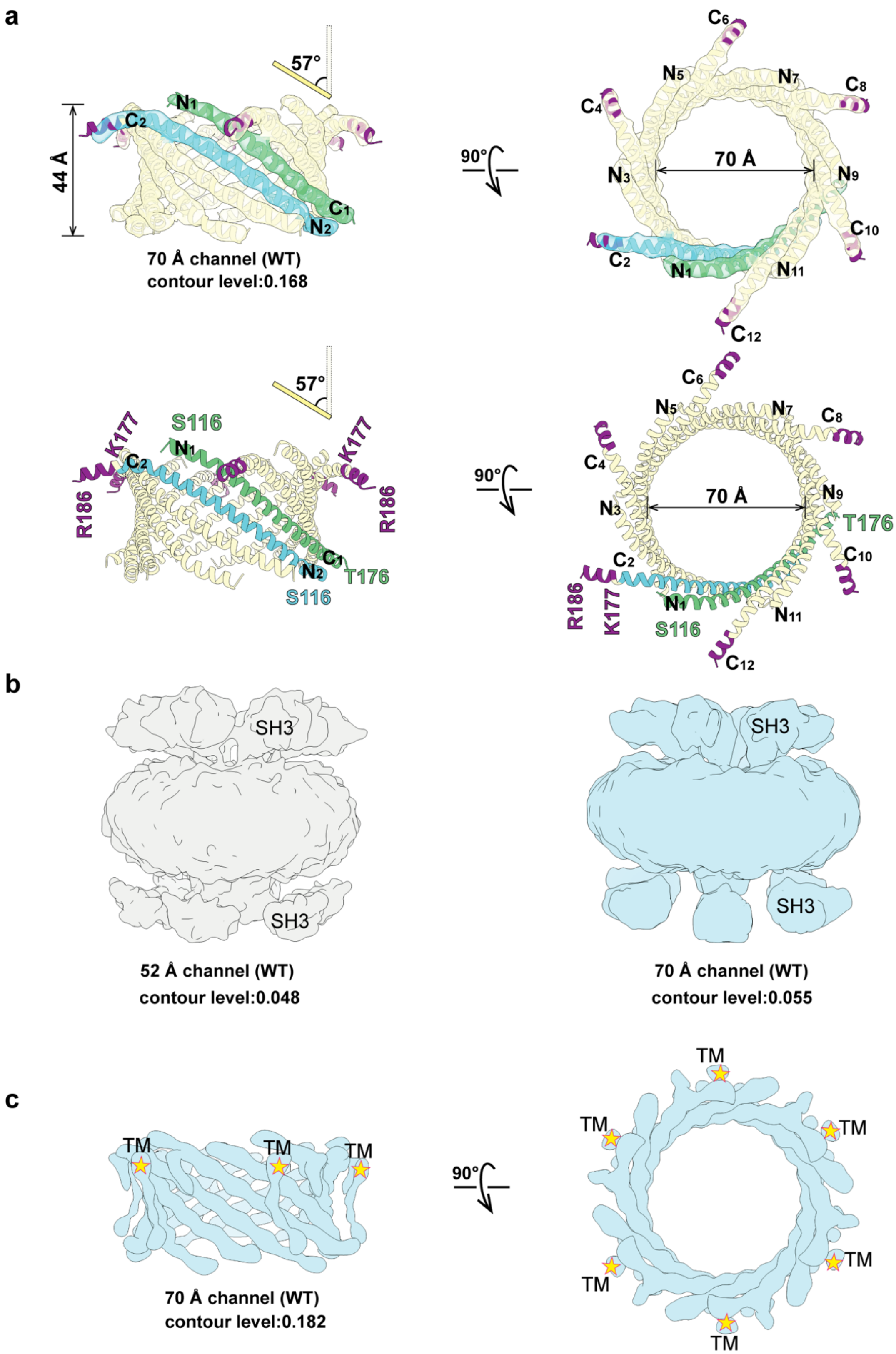

1072

1073

bioRxiv preprint doi: https://doi.org/10.64898/2026.10.23.761396; this version posted October 28, 2026. The copyright holder for this preprint (which was not certified by peer review) is the author/funder, who has granted bioRxiv a license to display the preprint in perpetuity. It is made available under a CC-BY 4.0 International license.

Extended Data Fig. 3 | Features in cryo-EM maps of human PEX13.

a, Semi-transparent density map of the ~70 Å channel in which residues 177-186 of the AH are visible (shown in purple). Two views are shown in the top panels, with a docked cartoon model based on AlphaFold3 (**Extended Data Fig. 4a**). The lower panels show the model only, with the additional C-terminal residues labeled. **b**, Density maps for the ~52 Å and ~70 Å channels at low contour level to visualize the SH3 domains. **c**, Density map for the ~70 Å channel. Putative density for the TM segments is indicated by a yellow star.

bioRxiv preprint doi: https://doi.org/10.64898/2026.10.23.761396; this version posted October 28, 2026. The copyright holder for this preprint (which was not certified by peer review) is the author/funder, who has granted bioRxiv a license to display the preprint in perpetuity. It is made available under a CC-BY 4.0 International license.

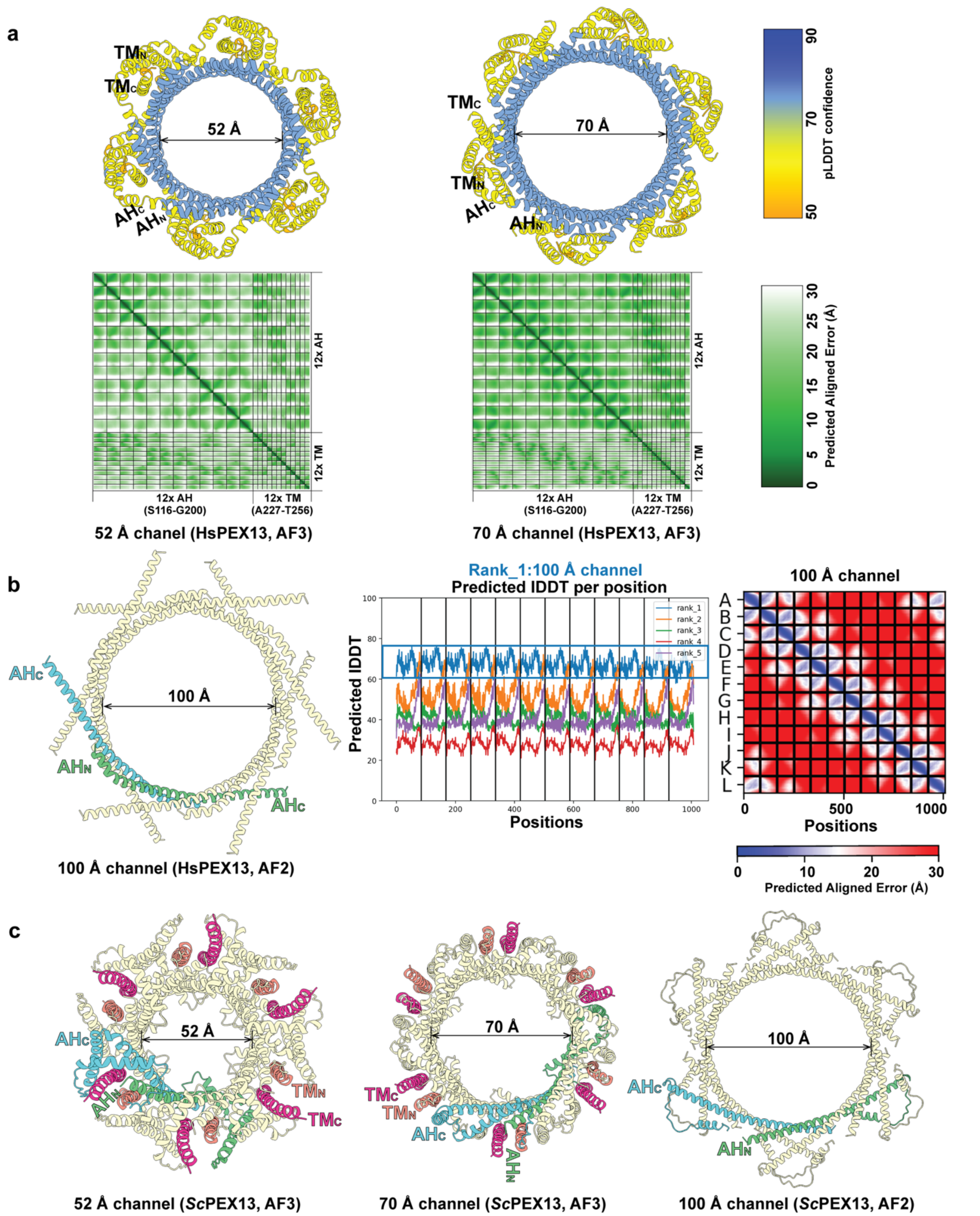

1081

1082

bioRxiv preprint doi: https://doi.org/10.64898/2026.10.23.761396; this version posted October 28, 2026. The copyright holder for this preprint (which was not certified by peer review) is the author/funder, who has granted bioRxiv a license to display the preprint in perpetuity. It is made available under a CC-BY 4.0 International license.

Extended Data Fig. 4 | Different channels predicted by AlphaFold.

a, AlphaFold3 (AF3)-predicted human PEX13 channels with pore diameters of ~52 Å and ~70 Å. The predictions were performed with 12 copies of the AH and 12 copies of the TM. The structure is colored according to the pLDDT confidence scale on the right. The lower panels show the predicted aligned error (Å), with the scale on the right. **b**, AlphaFold2 (AF2)-predicted human PEX13 channel with pore diameter of ~100 Å (left panel), based on rank 1 model that shows the highest values in the pLDDT plot (middle panel). The PAE plot is also shown (right panel), with the color scale at the bottom. **c**, AlphaFold-predicted *S. cerevisiae* PEX13 channels with pore diameters of ~52 Å, ~70 Å, and ~100 Å. The predictions of the smaller channels were done with 12 copies of AH (yellow, green and cyan) and 12 copies of TM (deep pink and salmon). The prediction of the largest channel was done with 12 AH copies only.

bioRxiv preprint doi: https://doi.org/10.64898/2026.10.23.761396; this version posted October 28, 2026. The copyright holder for this preprint (which was not certified by peer review) is the author/funder, who has granted bioRxiv a license to display the preprint in perpetuity. It is made available under a CC-BY 4.0 International license.

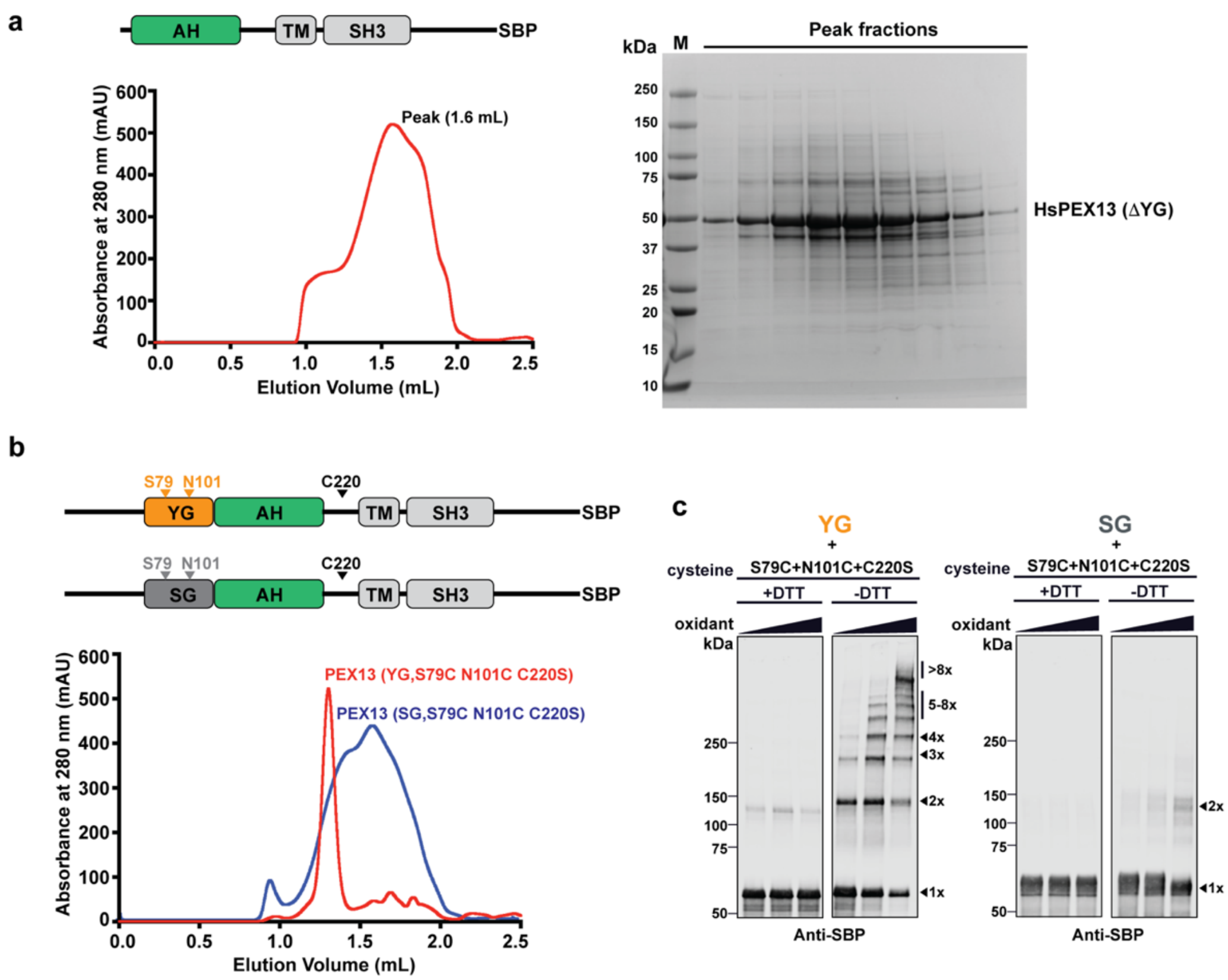

Extended Data Fig. 5 | The YG phase is required for the assembly of the PEX13 pore.

a, SBP-tagged human PEX13 lacking the YG domain (see scheme) was expressed in *P. pastoris*. The protein was solubilized in digitonin, purified with streptavidin beads, and subjected to SEC on a Superose 6 column (left panel). Note that the protein elutes at a lower molecular weight than the wild-type protein (1.6 mL versus 1.3 mL). The peak fractions were analyzed by SDS-PAGE and Coomassie blue staining (right panel). **b**, Human PEX13 with the wild-type YG domain or a mutant in which all tyrosines in the YG domain were mutated to serines (SG mutant) were expressed in *P. pastoris*. Both proteins also contained cysteines incorporated at positions 79 and 101 and had their endogenous cysteine at position 220 changed to serine. The proteins were purified as in (A). Note that the SG mutant (blue curve) elutes in SEC as a broad peak later than the protein with wild-type YG domain (red curve). **c**, The purified proteins were treated with Cu-phenanthroline (oxidant), and analyzed by nonreducing (-DTT) or reducing (+DTT) SDS-PAGE and immunoblotting with SBP antibodies. The number of crosslinked PEX13 molecules in the bands is indicated on the right.

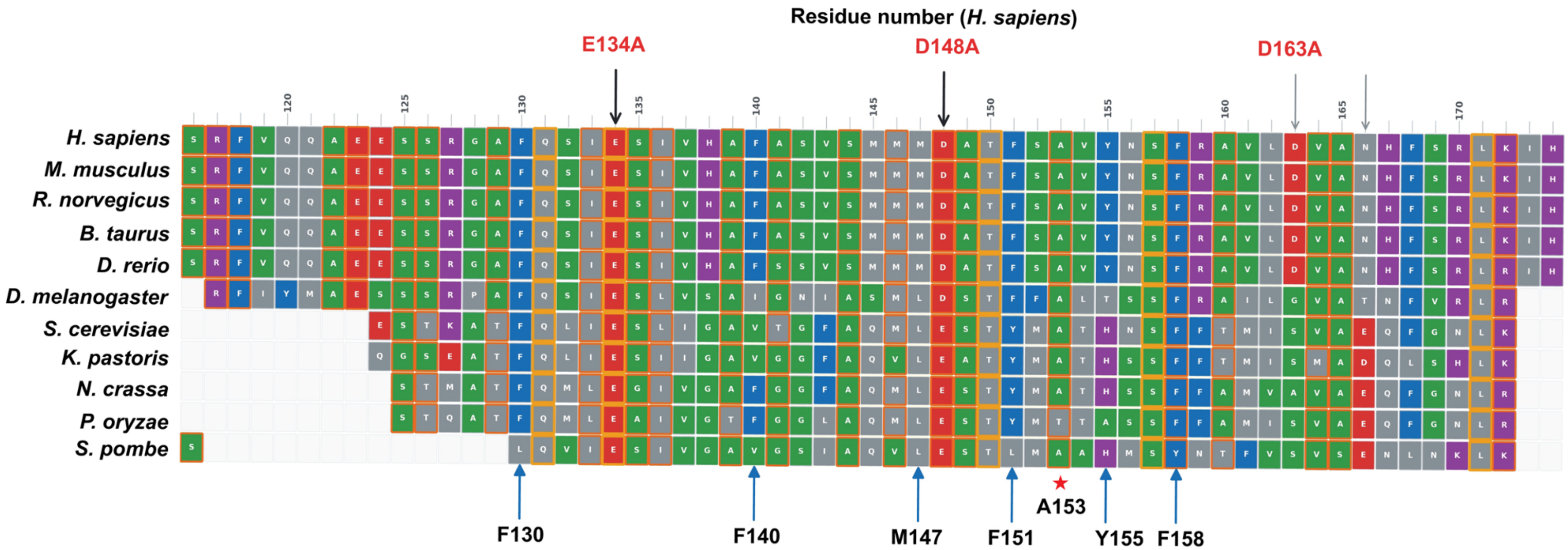

Extended Data Fig. 6 | Amino acid sequence conservation of the AH.

Sequence alignment of the AHs from organisms representative of different eukaryotic phyla, performed with the help of Claude. The colors group residues by chemical type: red, acidic (D, E); blue, aromatic (F, W, Y, H); purple, basic (K, R); green, small/hydrophilic (A, G, V, S, T); grey, all others. The three highlighted conserved acidic residues were changed to alanines in human PEX13. The corresponding mutations in *S. cerevisiae* PEX13 included the two universally conserved acid residues, as well as a glutamate three residues downstream of D163 that is conserved in some organisms (indicated by an arrow). The blue arrows show the position of amino acids in the constant interface between AHs of human PEX13. The red star indicates the reference residues in the variable interface.

bioRxiv preprint doi: https://doi.org/10.64898/2026.10.23.761396; this version posted October 28, 2026. The copyright holder for this preprint (which was not certified by peer review) is the author/funder, who has granted bioRxiv a license to display the preprint in perpetuity. It is made available under a CC-BY 4.0 International license.

bioRxiv preprint doi: https://doi.org/10.64898/2026.10.23.761396; this version posted October 28, 2026. The copyright holder for this preprint (which was not certified by peer review) is the author/funder, who has granted bioRxiv a license to display the preprint in perpetuity. It is made available under a CC-BY 4.0 International license.

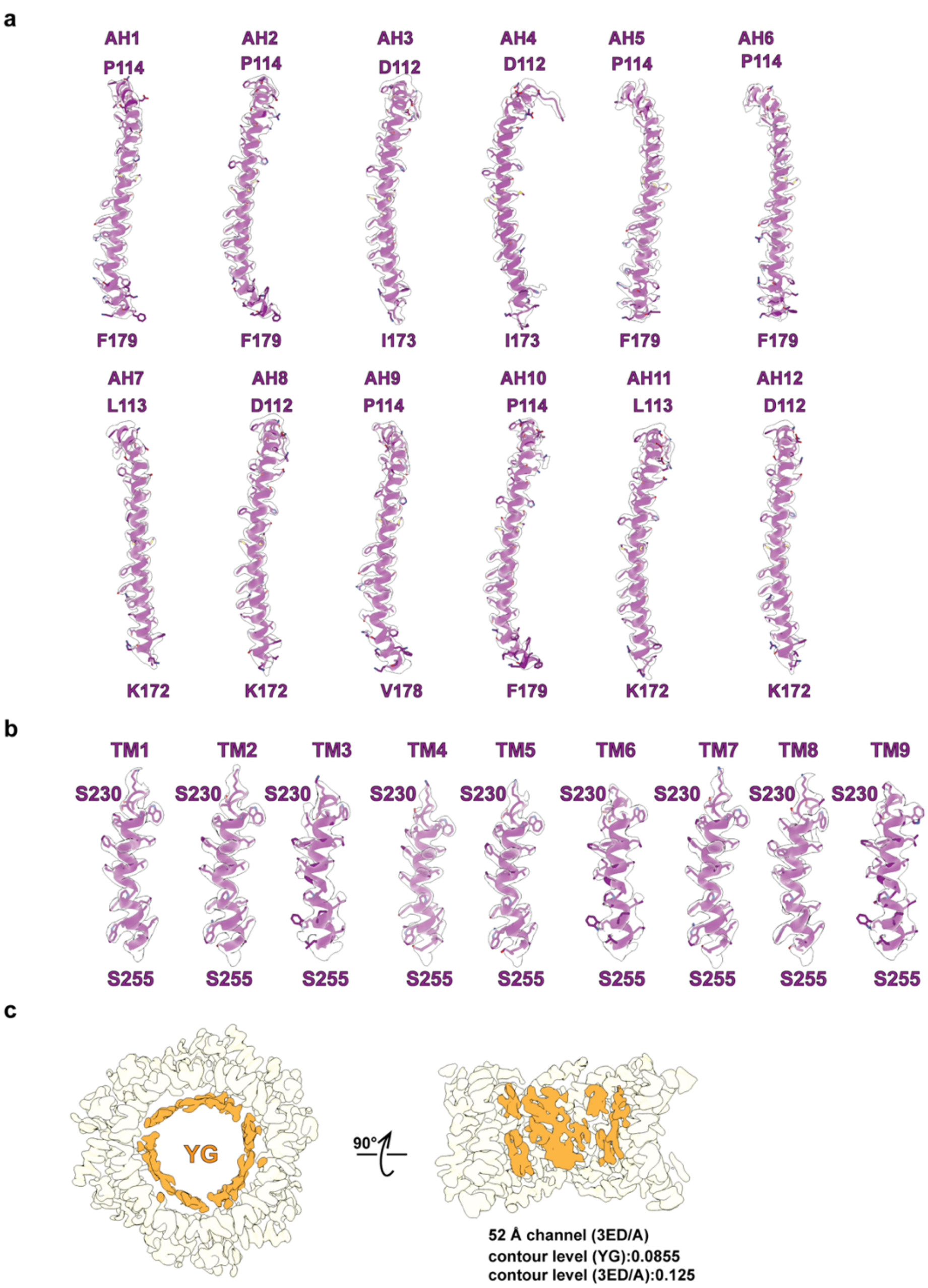

Extended Data Fig. 7 | Fit of the model into the density map of the 3ED/A mutant channel.

a, Shown are the density maps for the 12 AHs and the models built into them.

b, As in (A), but for the TMs.

c, Visualization of the YG domains (in orange) at low contour level. The density for the AHs is shown in yellow at a higher threshold level.

bioRxiv preprint doi: https://doi.org/10.64898/2026.10.23.761396; this version posted October 28, 2026. The copyright holder for this preprint (which was not certified by peer review) is the author/funder, who has granted bioRxiv a license to display the preprint in perpetuity. It is made available under a CC-BY 4.0 International license.

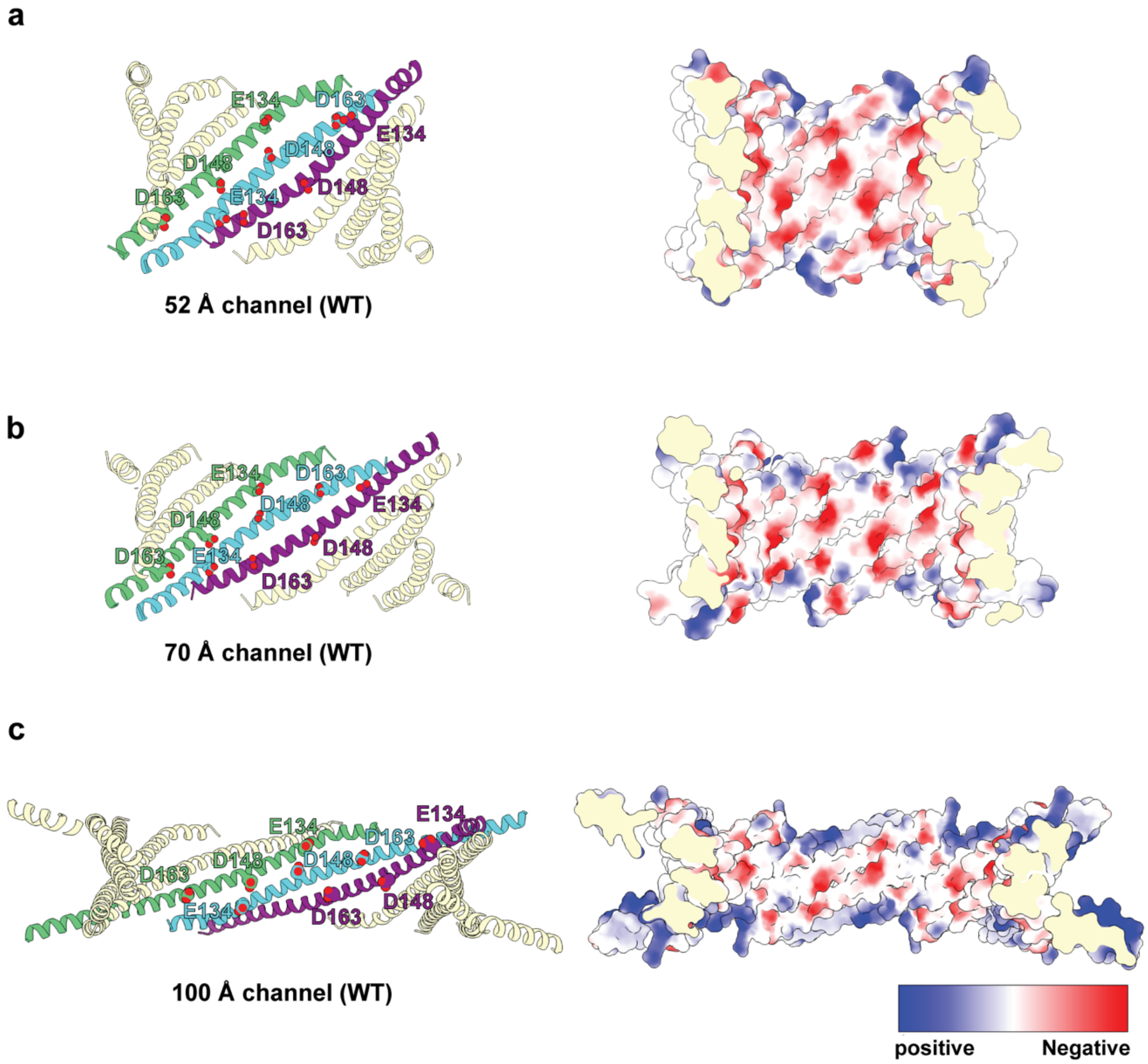

Extended Data Fig. 8 | Positions of acidic residues in the human PEX13 channels.

a, Position of the three conserved acidic residues in the ~52 Å channel. The left panel shows a cartoon model with the acidic residues marked in three neighboring AHs. The right model shows a space-filling model with the inside of the pore shown in a cut-away view. The surface is colored according to the electrostatic potential of the amino acids (scale at the bottom). **b**, As in **a**, but for the ~70 Å channel. **c**, As in **a**, but for the ~100 Å channel. Note that the negative charges are most concentrated in the ~52 Å channel, which might facilitate the conversion into the active channels by mutual repulsion.

bioRxiv preprint doi: https://doi.org/10.64898/2026.10.23.761396; this version posted October 28, 2026. The copyright holder for this preprint (which was not certified by peer review) is the author/funder, who has granted bioRxiv a license to display the preprint in perpetuity. It is made available under a CC-BY 4.0 International license.

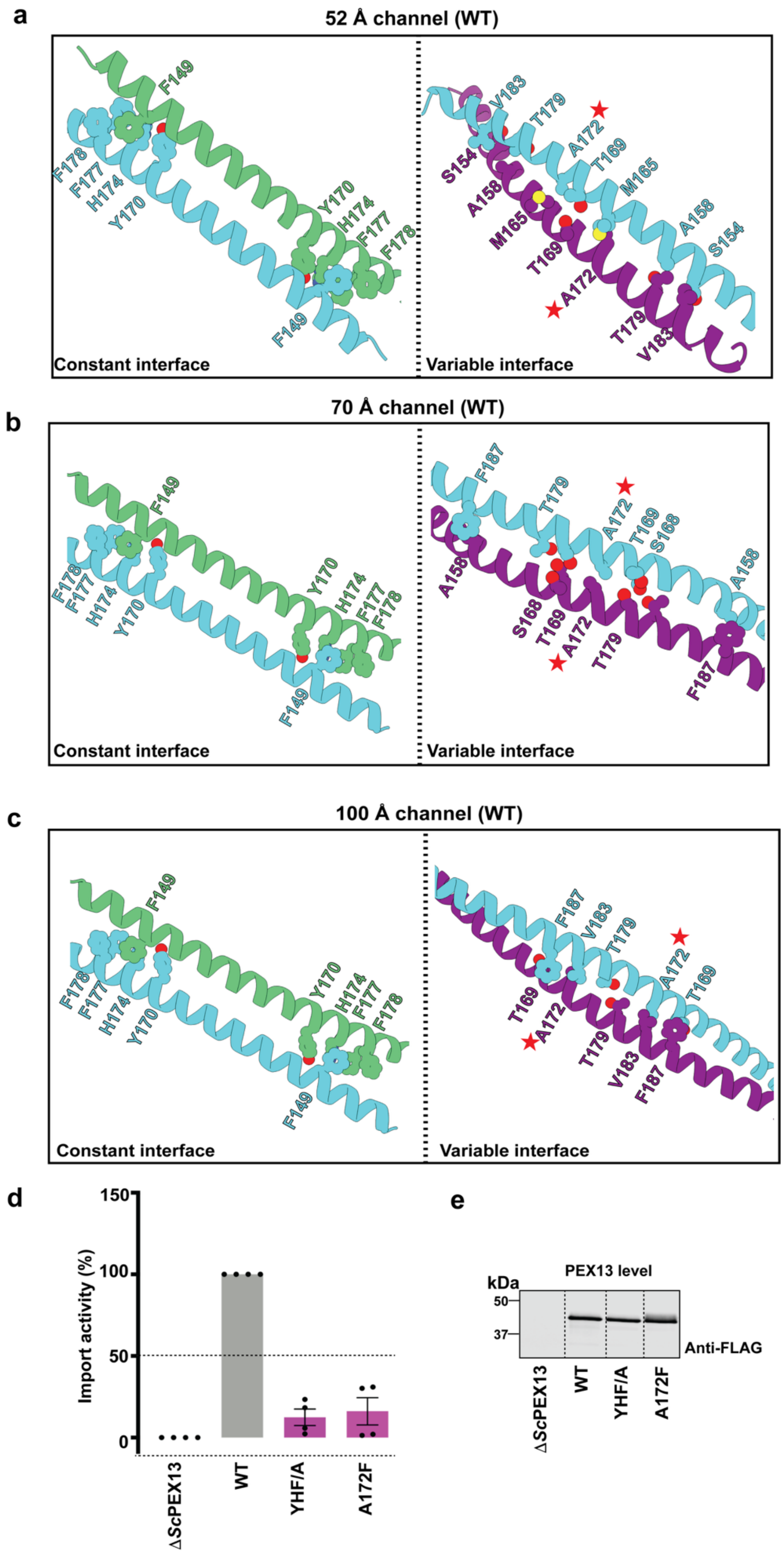

1137

bioRxiv preprint doi: https://doi.org/10.64898/2026.10.23.761396; this version posted October 28, 2026. The copyright holder for this preprint (which was not certified by peer review) is the author/funder, who has granted bioRxiv a license to display the preprint in perpetuity. It is made available under a CC-BY 4.0 International license.

Extended Data Fig. 9 | Constant and variable AH interfaces for the predicted *S. cerevisiae* channels.

a, Constant and variable interfaces of the AlphaFold-predicted ~52Å channel. Interacting amino acids are labeled and shown with side chains. Hetero-atoms in the side chains are shown in different colors. Note that the constant interface contains aromatic residues, and the variable interface amino acids with small side chains, as in mammals (**Fig. 4c**). A position in the variable interface is marked by a star to facilitate the comparison with the other channel states shown in **b** and **c**. **b**, As in **a**, but for the predicted ~70 Å channel. **c**, As in **a**, but for the predicted ~100 Å channel. **d**. FLAG-tagged wild-type (WT) *S. cerevisiae* PEX13 or the indicated mutants were expressed in *S. cerevisiae* cells lacking endogenous PEX13 *(Δpex13*). The YHF/A mutant carries alanine mutations of aromatic residues in the constant AH interface (Y170A, H174A, and F177A (corresponding to F151, Y155, and F158 in HsPEX13). The A172F mutant (corresponding to A153 in HsPEX13) carries a mutation in the variable interface. Peroxisomal protein import was measured with an engineered pathway that generates a pigment when import is impaired. Import activity is plotted relative to WT PEX13 (mean ± standard error of 4 experiments). **e**. The expression level of PEX13 in the experiments in **d** was analyzed by SDS-PAGE and immunoblotting with FLAG antibodies.

bioRxiv preprint doi: https://doi.org/10.64898/2026.10.23.761396; this version posted October 28, 2026. The copyright holder for this preprint (which was not certified by peer review) is the author/funder, who has granted bioRxiv a license to display the preprint in perpetuity. It is made available under a CC-BY 4.0 International license.

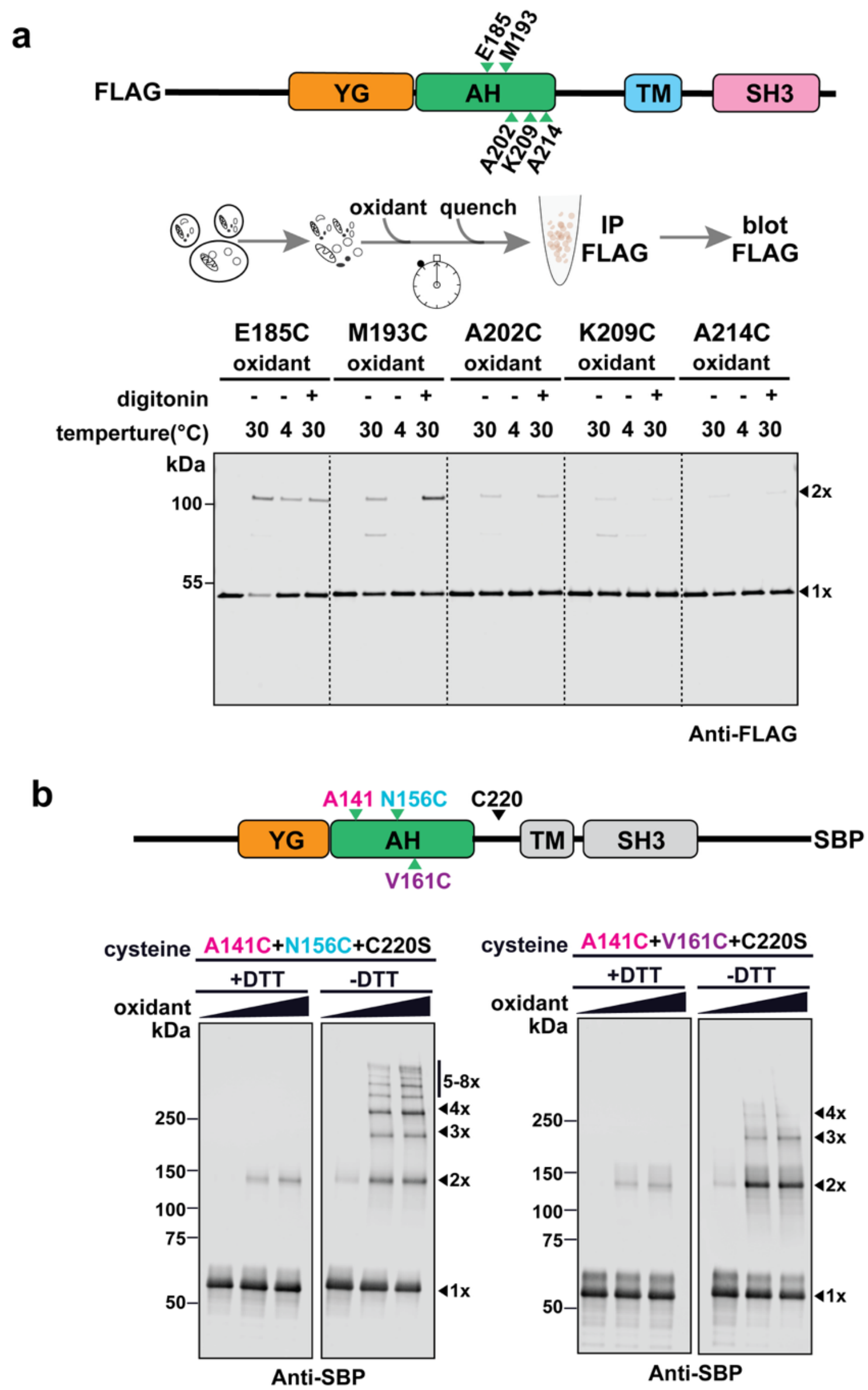

1169

1170 **Extended data Fig. 10 | Disulfide crosslinking of yeast and human PEX13.**

1171 **a**. FLAG-tagged yeast PEX13 variants containing single cysteines at the indicated positions
1172 were expressed in *S. cerevisiae* cells. The positions chosen are either in the AH (E185,
1173 M193, A202) or in the following region (K209, A214). These positions are not expected to be
1174 close to the same position in a neighboring AH and thus serve as controls for the
1175 experiment shown in **Fig. 4a**. **b**, SBP-tagged human PEX13 variants containing two
1176 cysteines were expressed in *P. pastoris* cells, purified with streptavidin beads, and treated
1177 with an oxidant. The samples were analyzed by reducing (+DTT) or non-reducing (-DTT)
1178 SDS-PAGE, followed by immunoblotting with SBP antibodies. One of the cysteines was
1179 placed at position A141 in the constant interface of the AHs, the other at position N156 or
1180 V161 in the variable interface.

bioRxiv preprint doi: https://doi.org/10.64898/2026.10.23.761396; this version posted October 28, 2026. The copyright holder for this preprint (which was not certified by peer review) is the author/funder, who has granted bioRxiv a license to display the preprint in perpetuity. It is made available under a CC-BY 4.0 International license.

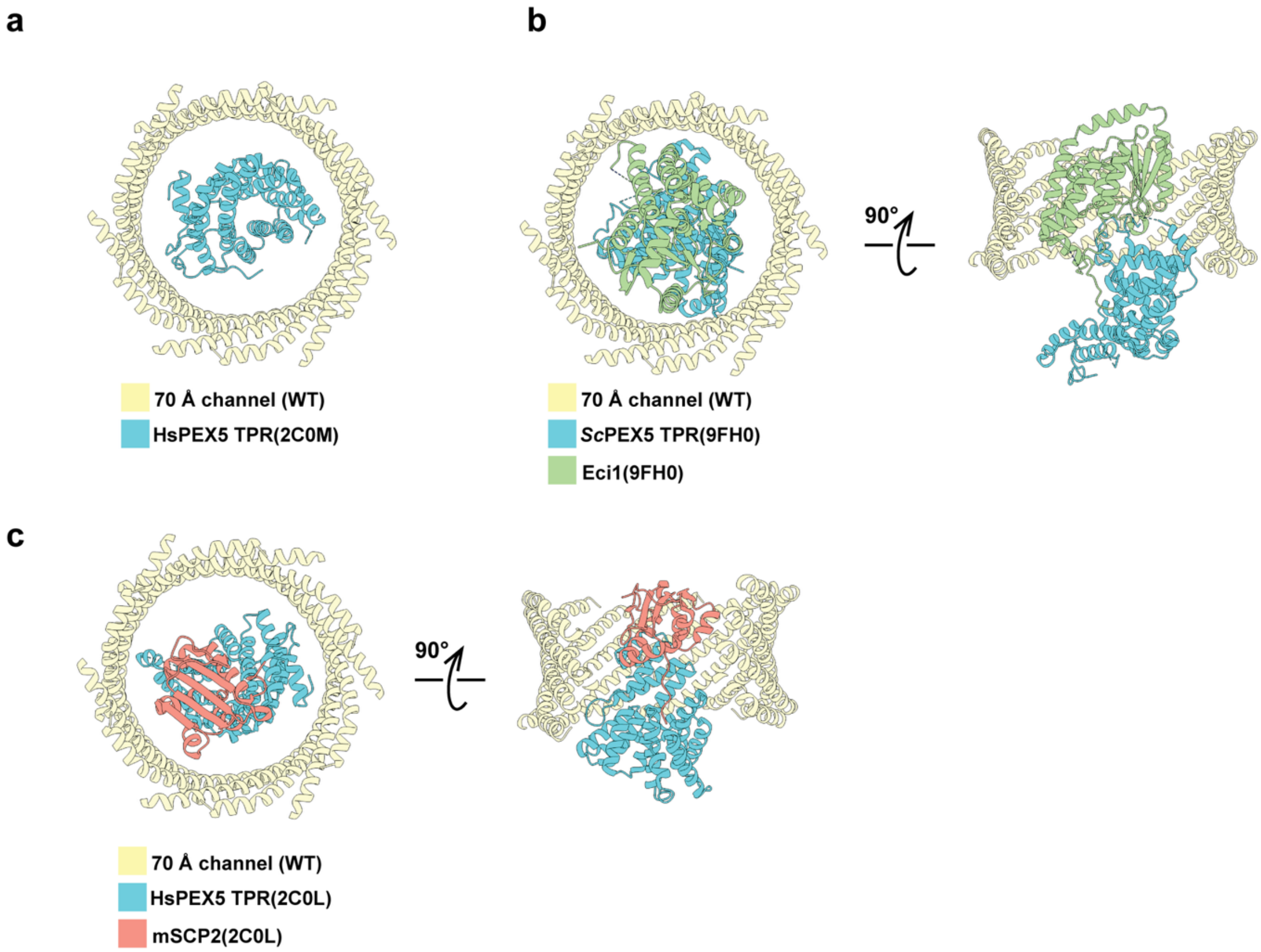

Extended Data Fig. 11 | Fitting of receptor and small cargo into the ~70 Å channel.

a, The TPR domain of human PEX5 (HsPEX5) (PDB code: 2C0M) was placed into the ~70 Å channel. Shown is a top view of cartoon models. **b**, As in **a**, but with the complex of the TPR domain of *S. cerevisiae* PEX5 and the cargo protein Eci1 (PDB code: 9FH0). Shown is a top view (left panel) and a side view with the front part of the channel removed for clarity (right panel). **c**, As in **b**, but for the complex of the TPR domain of HsPEX5 and the cargo mSCP2 (PDB code: 2C0L).

bioRxiv preprint doi: https://doi.org/10.64898/2026.10.23.761396; this version posted October 28, 2026. The copyright holder for this preprint (which was not certified by peer review) is the author/funder, who has granted bioRxiv a license to display the preprint in perpetuity. It is made available under a CC-BY 4.0 International license.

Extended Data Table. 1 | Cryo-EM data collection, refinement and validation statistics in this study

	52Å import channel (WT) (EMDB:78756)	70Å import channel (WT, no 177-186) (EMB:78762)	70Å import channel (WT, 177-186) (EMB: 78763)	52Å import channel (3ED/A) (EMDB: 78822) (PDB: 38HQ)
**Data collection and processing**				
Microscope	Krios	Krios	Krios	Krios
Magnification	81,000	81,000	81,000	81,000
Voltage (kV)	300	300	300	300
Total electron exposure (e–/Å^2)	50	50	50	50
Defocus range (μm)	-1.0 to -2.5	-1.0 to -2.5	-1.0 to -2.5	-1.0 to -1.8
Pixel size (Å/pixel)	1.06	1.06	1.06	0.857
Symmetry imposed	C6	C6	C6	C3
Initial particle images (no.)	1,561,361	1,561,361	1,561,361	3,213,428
Final particle images (no.)	36,627	50,745	49,051	75,550
Resolution (global, Å)				
FSC 0.5	7.2/6.7	7.2/6.6	7.5/6.8	3.9/3.4
(unmasked/masked)	6.6/6.1	6.5/6.2	7.0/6.2	3.4/2.8
FSC 0.143 (unmasked/masked)				
**Model Refinement**				
Initial model used (PDB code)				
Model resolution (Å)				2.94
FSC threshold				0.5
Map resolution range (Å)	5.0 to 8.0	5.0 to 8.0	5.0 to 8.0	2.5 to 5.0
Map sharpening B factor (Å2)	-705.4	-707.4	-796.9	-92.1
Model composition				
Non-hydrogen atoms				7839
Protein residues				1003
Ligands				0
B factors (Å^2)				
Protein residues				79.40
Ligands				---
R.m.s. deviations				
Bond lengths (Å)				0.002
Bond angles (°)				0.403
Validation				
MolProbity score				1.27
Clashscore				5.03
Poor rotamers (%)				0.36
Ramachandran plot				
Favored (%)				99.27
Allowed (%)				0.73
Disallowed (%)				0.00

bioRxiv preprint doi: https://doi.org/10.64898/2026.10.23.761396; this version posted October 28, 2026. The copyright holder for this preprint (which was not certified by peer review) is the author/funder, who has granted bioRxiv a license to display the preprint in perpetuity. It is made available under a CC-BY 4.0 International license.

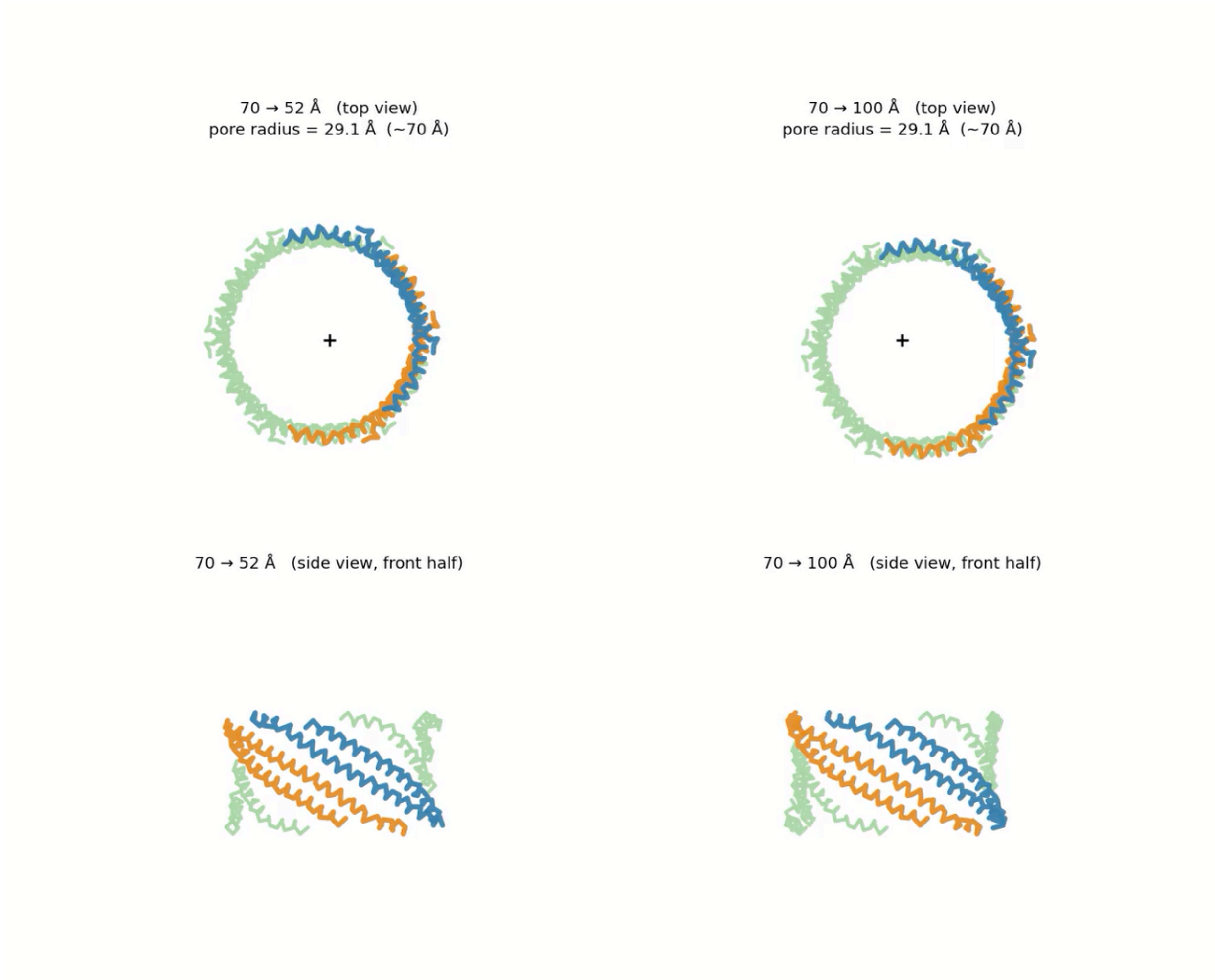

Extended Data Movie 1 | Iris-like mechanism of channel conversion.

Shown is the morphing between the PDB files of the ~52 Å and the ~70 Å channels (left panels) and between the ~70 Å and ~100 Å channels (right panels), generated by Claude. The upper panels show cartoon models of the AHs, viewed down onto the membrane (top views), and the lower panels show side views with AHs in the back removed for clarify. Two dimers connected by a constant interface are shown in orange and blue. Note that the dimers slide relative to one another like an iris, generating the different tilt angles, pore diameters, and pore heights. In the video, all AHs move simultaneously, but in reality the transitions could be more complicated.
